# The catalytic performance of Ru–NHC alkylidene complexes: PCy_3_ versus pyridine as the dissociating ligand

**DOI:** 10.3762/bjoc.6.136

**Published:** 2010-12-15

**Authors:** Stefan Krehl, Diana Geißler, Sylvia Hauke, Oliver Kunz, Lucia Staude, Bernd Schmidt

**Affiliations:** 1 Universität Potsdam, Institut für Chemie, Organische Synthesechemie, Karl-Liebknecht-Straße 24–25, D-14476 Golm, Germany

**Keywords:** homogeneous catalysis, *N*-heterocyclic carbenes, olefin metathesis, pyridine ligand, Ruthenium carbene complexes

## Abstract

The catalytic performance of NHC-ligated Ru-indenylidene or benzylidene complexes bearing a tricyclohexylphosphine or a pyridine ligand in ring closing metathesis (RCM), cross metathesis, and ring closing enyne metathesis (RCEYM) reactions is compared. While the PCy_3_ complexes perform significantly better in RCM and RCEYM reactions than the pyridine complex, all catalysts show similar activity in cross metathesis reactions.

## Introduction

Over the past two decades, the olefin metathesis reaction became one of the most important C–C-bond forming reactions in organic synthesis [1]. The elucidation of the crucial role of metal carbenes by Chauvin [2] and the development of stable and defined precatalysts for homogeneously catalyzed reactions by Schrock [3] and Grubbs [4] paved the way for this development. Since then, molybdenum- [5] and ruthenium-based [6] catalysts have experienced extensive further developments and improvements. Due to their robustness towards air and moisture, and their comparatively low sensitivity towards functional groups, Ru-carbene complexes have attracted a particularly high degree of attention and “numerous variations on a theme by Grubbs”, as so accurately phrased by Fürstner [7], have been published. In the early stages of catalyst evolution improved methods for the introduction of the alkylidene ligand were the main focus. Thus, the original version of first generation Grubbs’ catalyst (**A**) [8], in which the alkylidene moiety originates from 2,2-diphenylcyclopropene, was soon replaced by the second version **B** [9], since the benzylidene ligand is more conveniently available from phenyldiazomethane. The obvious disadvantages of handling non-stabilized diazo compounds stimulated investigations into the use of propargylic alcohols as alkylidene precursors [10], which resulted in the synthesis of a first generation analogue **C** with an indenylidene ligand [11–12]. A landmark in the evolution of Ru-metathesis catalysts was the introduction of alkylidene complexes bearing *N*-heterocyclic carbenes (NHC) [13–15], in particular complex **D**, which became known as second generation Grubbs’ catalyst [16], and the Umicore M2 catalyst **E** [17–18]. The ligation of strongly σ-donating NHC’s leads to an improvement of catalytic activity, which sometimes equals the activity of Mo-based catalysts while maintaining the general robustness and tolerance towards several functional groups [19]. A third major topic in catalyst development has been the variation of the dissociating “placeholder”-ligand. In this respect, the introduction and further improvement of hemilabile benzylidene ligands by Hoveyda [20], Grela[21] and Blechert [22–23] have been important achievements. These phosphine-free catalysts of the general type **F** sometimes show higher activities due to enhanced catalyst lifetimes and have often been applied in cross metathesis reactions [24]. An alternative approach to phosphine-free Ru-metathesis catalysts uses pyridines as placeholders. Originally, complex **G** was synthesized as a precursor for mixed NHC-phosphine complexes other than **D** [25–28] or, very recently, for the synthesis of Ru-alkylidenes with two different NHC ligands [29]. Comparatively little information is available concerning the catalytic activity of these pyridine-NHC complexes. They were found to initiate ring opening metathesis polymerization reactions very rapidly [30–31] and show a certain preference for cross metathesis [32–35]. If the pyridine ring is attached to the alkylidene ligand [36], a latent catalyst results which might be a particularly useful property for metathesis polymerization reactions [37]. More recently, Ru-indenylidene complexes bearing one or two pyridine ligands were also synthesized [38–39], with the commercially available Umicore M31 catalyst **H** being a particularly interesting example. This catalyst is available from the M2 complex by ligand substitution [18] and shows high activity in living ROMP [40], whereas the reactivity in some RCM and CM reactions appears to be diminished [18,41].

**Figure 1:**
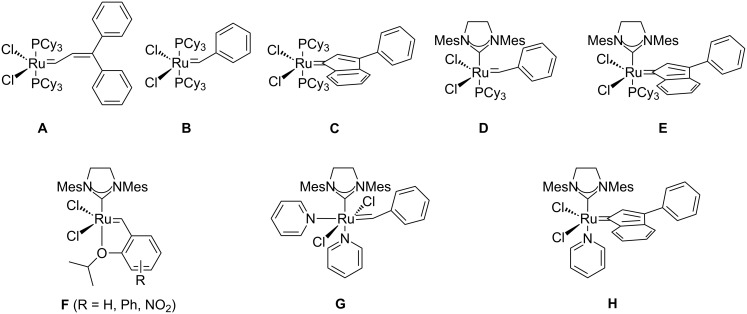
Ru-based metathesis catalysts.

During our research directed at the development of novel metathesis-non metathesis reaction sequences [42] catalyzed by a single site catalyst and initiated by organometallic transformations in situ, we became interested in the use of phosphine-free catalyst **H**. Unfortunately, only limited data is available concerning the catalytic efficiency of this catalyst in small molecule metathesis, in particular in comparison to the established phosphine containing catalysts **D** and **E** (Figure 1).

In this contribution, representative ring closing olefin, ring closing enyne and cross metathesis reactions of indenylidene complexes **E** and **H** and benzylidene complex **D** are compared.

## Results and Discussion

### Effects of solvent and catalyst loading

**Scheme 1:**
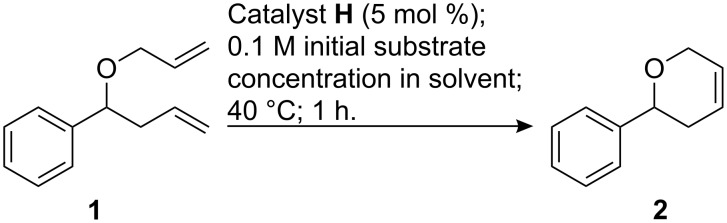
RCM of an allyl ether catalyzed by catalyst **H**.

As a test reaction, we initially investigated the ring closing metathesis of allyl ether **1** to dihydropyran **2** (Scheme 1). To this end, conversion to the desired product in the presence of 5 mol % of catalyst **H** was determined after a reaction time of one hour at a slightly elevated temperature in seven different solvents, under otherwise identical conditions.

**Figure 2:**
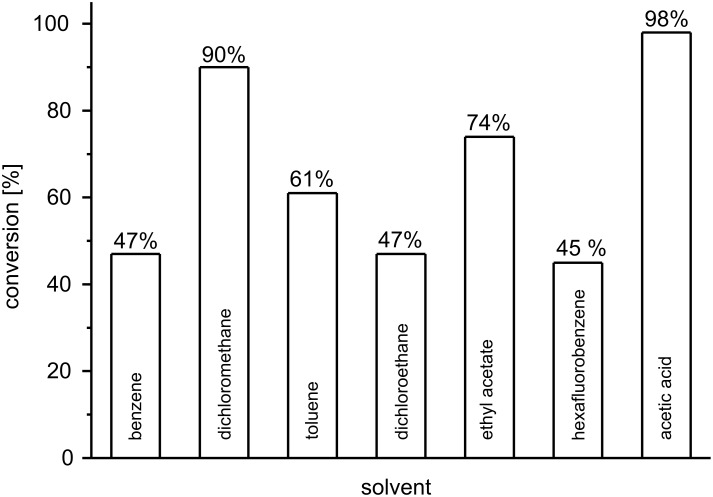
Solvent screening for the RCM of **1** catalyzed by **H** (standardized conditions as denoted in Scheme 1).

The results are summarized in Figure 2. Benzene, toluene, dichloromethane and 1,2-dichloroethane are commonly used solvents in Ru-catalyzed RCM reactions. However, for catalyst **H** only in dichloromethane was high conversion to the desired RCM product observed. In hexafluorobenzene, which has recently been shown to give highly impressive results, even in difficult metathesis reactions catalyzed by **D**, **E**, or **F** [43–44], the rate of conversion to dihydropyran **2** is quite similar to benzene or dichloroethane. Polar and, in particular, protic solvents would normally be considered inappropriate for metathesis reactions, because catalyst inhibition or degradation to Ru-hydrides might occur [45–47]. Nevertheless, such solvents have previously been investigated and useful results were obtained for esters [48] and – even more surprising – for acetic acid [49]. Therefore, we used ethyl acetate and acetic acid for our test reaction. While ethyl acetate gave a conversion of 75%, which is better than most of the classical solvents, nearly quantitative conversion to the RCM product was observed in acetic acid. Presumably, the pyridine ligand is protonated under these conditions which would result in a higher amount of the catalytically active 14-electron species. This interpretation is corroborated by the kinetic data obtained by Adjiman, Taylor et al. in their original investigation on solvent effects [49].

**Figure 3:**
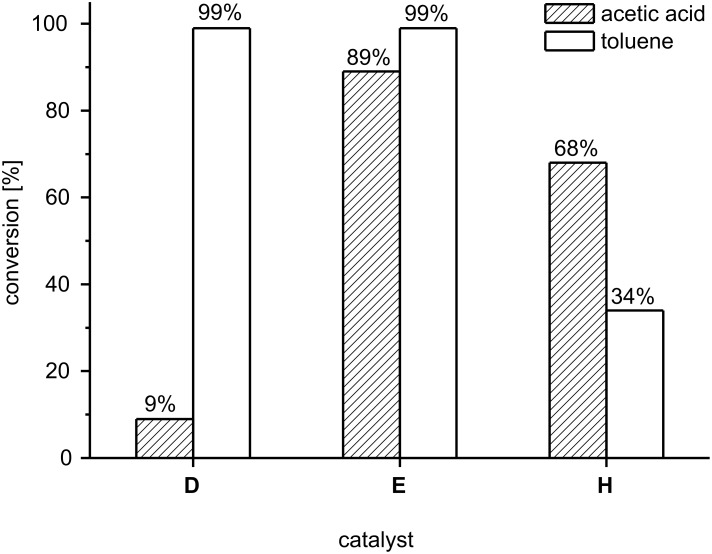
Comparison of catalysts **D**, **E** and **H** in toluene and acetic acid (standardized conditions as denoted in Scheme 1).

Next, we were interested to see how the performance of pyridine containing catalyst **H** compares with the more established phosphine complexes **D** and **E**. The test reaction depicted in Scheme 1 was therefore repeated in toluene and in acetic acid with a significantly lower catalyst loading, because we assumed that the highly active catalysts **D** and **E** would otherwise lead to full conversion in extremely short time (Figure 3).

Thus, with 1.0 mol % of catalyst under conditions identical to those given in Scheme 1, a quantitative conversion to the dihydropyran **2** was observed for both catalysts **D** and **E** in toluene. In accord with the results summarized in Figure 2, catalyst **H** gave only 34% conversion in this solvent after one hour, suggesting that initiation was rather slow. In acetic acid, the analogous phosphine containing indenylidene catalyst **E** displayed a slightly reduced conversion of 89%, which might be attributed to slow catalyst deactivation, whereas pyridine complex **H** showed significantly enhanced activity in acetic acid, with a conversion of 68% after one hour. This result suggests that by switching from toluene to acetic acid the balance of catalyst deactivation and enhanced initiation is shifted to the deactivation side for phosphine catalyst **E**, and to the enhanced initiation side for pyridine catalyst **H**. The most remarkable result from this set of experiments, however, is a collapse of conversion if benzylidene catalyst **D** is used in acetic acid. With **D**, a reproducible conversion of only 9% to the dihydropyran **2** was observed, compared to 89% conversion with the analogous indenylidene complex **E**. This result seems to contradict the observations by Adjiman, Taylor et al. who reported preparatively useful conversions and yields for second generation Grubbs’ catalyst **D** in acetic acid [49], however, their studies were conducted at ambient temperature and for different substrates, while our experiments were conducted at 40 °C. It is possible that our result suggests a higher robustness of indenylidene catalyst **E** compared to benzylidene catalyst **D**, at least under these rather unusual conditions.

**Figure 4:**
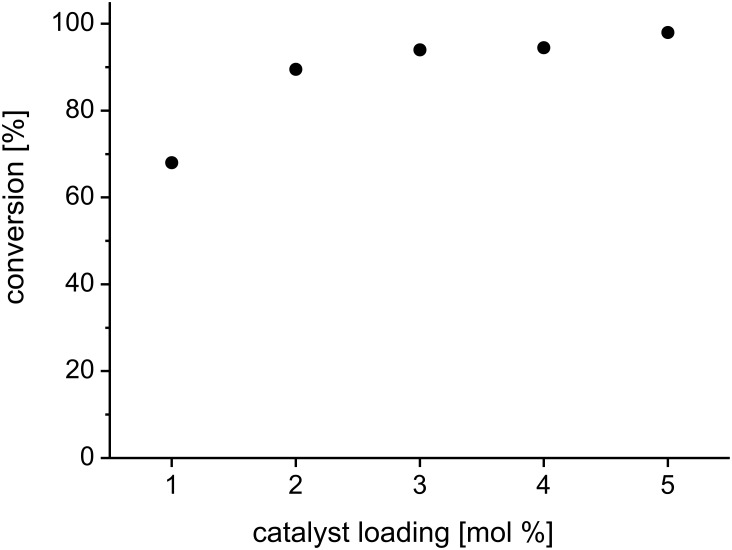
Conversion vs catalyst loading for the RCM of **1** in acetic acid (standardized conditions as denoted in Scheme 1).

To further evaluate the catalytic performance of pyridine catalyst **H** in acetic acid, we next wanted to determine the minimum amount of catalyst required to obtain a preparatively useful (>90%) rate of conversion. Consequently, it was demonstrated that, instead of using the conditions noted in Scheme 1, a catalyst loading of 2 mol % was sufficient to achieve a 92% conversion within one hour (Figure 4).

### Ring closing metathesis of acrylates

**Scheme 2:**
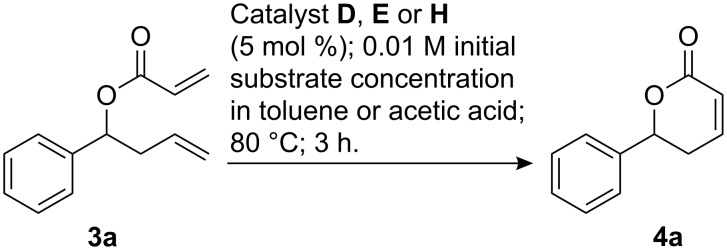
Catalyst screening for RCM of acrylate **3a**.

Having established the beneficial effect of acetic acid for the ring closing metathesis reaction of allyl ether **1** catalyzed by **H**, we were interested to see if a similar effect exists for other substrates. Therefore, acrylate **3a** was subjected to the conditions of a ring closing metathesis reaction (Scheme 2 and Table 1). Not unexpectedly [50], significantly reduced initial substrate concentrations were required for useful rates of conversion to the desired α,β-unsaturated lactone **4a**. It transpired, that preparatively useful yields could only be obtained with the phosphine containing catalysts **D** and **E** in toluene (Table 1, entries 1 and 2), whereas pyridine complex **H** gave a conversion of approximately 65% and an isolated yield of 41% of lactone **4a** in this solvent (Table 1, entry 3). In contrast to the ring closing metathesis of allyl ether **1**, the rate of conversion decreased significantly when catalyst **H** was used in acetic acid (Table 1, entry 4). Notably, the ^1^H NMR-spectra of the crude reaction mixtures did not indicate the presence of any decomposition products, thus, even the comparatively labile acrylate **3** appears to be quite stable in acetic acid at elevated temperatures for several hours.

**Table 1:** RCM of acrylate **3a**.

Entry	Solvent	Catalyst^a^	Ratio **4a**:**3a**^b^	Yield of **4a**

1	toluene	**D**	>15:1	80%
2	toluene	**E**	>15:1	92%
3	toluene	**H**	10:5	41%
4	acetic acid	**H**	10:17	—^c^

^a^A catalyst loading of 5 mol % was used in all experiments. ^b^Ratio of product to starting material was determined from the ^1^H NMR-spectrum of the crude reaction mixture after three hours at 80 °C. ^c^Yield was not determined due to low conversion.

**Figure 5:**
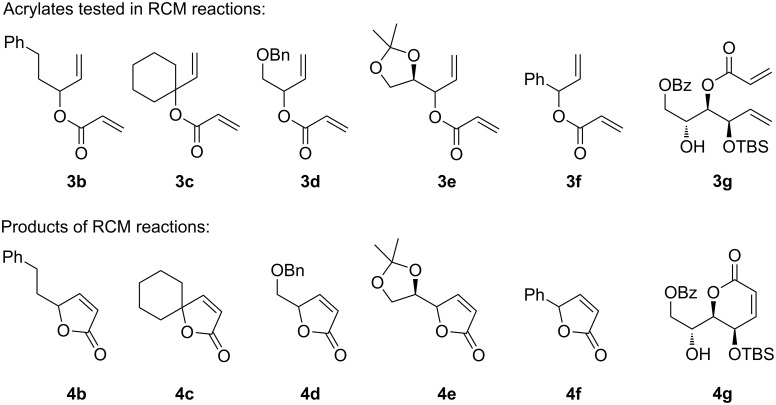
Acrylates **3** and their RCM products **4**.

Because acetic acid did not lead to the expected improvement in acrylate metathesis reactions, only toluene was tested as a solvent in further experiments (Figure 5 and Table 2). Toluene was preferred over dichloromethane, because reactions are more conveniently conducted at elevated temperatures in this solvent.

The acrylates **3** investigated in ring closing metathesis reactions with catalysts **D**, **E** and **H** are listed in Figure 5, together with the resulting unsaturated lactones **4**. Results for the ring closing metathesis of acrylates **3a**–**g** are summarized in Table 2. Lactones **4b**–**f** [50] are accessible in preparatively useful yields with catalyst loadings of 2.5 mol % to 5.0 mol % if catalysts **D** and **E** are used. Conversions observed with catalyst **H** under otherwise identical conditions are significantly lower and yields exceed 50% only in few cases such as **4b**, **4e** and **4f**. A particularly difficult substrate for olefin metathesis reactions is acrylate **3g**, which has recently been used by us as an intermediate in the synthesis of the natural product (–)-cleistenolide [51]. Acrylate **3g** requires very high dilution, the addition of phenol as a rate accelerating additive [52], a rather high catalyst loading of 10 mol % and an even higher reaction temperature. Under these conditions, the best product to substrate ratio and the best isolated yield was obtained with the indenylidene complex **E** (Table 2, entry 6).

**Table 2:** RCM reactions of acrylates to unsaturated lactones.^a^

Entry	**3**	**4**	*c*/mol·L^−1^	Catalyst loading	Ratio^b^ **4**:**3** (isolated yields) for		

					**D**	**E**	**H**

1	**3b**	**4b**	0.02	2.5 mol %	> 20:1 (86%)	>20:1 (90%)	1.8:1 (52%)
2	**3c**	**4c**	0.02	2.5 mol %	>20:1 (75%)	>20:1 (85%)	2.1:1 (43%)
3	**3d**	**4d**	0.02	2.5 mol %	7.1:1 (79%)	3.3:1 (41%)	0.6:1 (27%)
4	**3e**	**4e**	0.01	5.0 mol %	>10:1 (59%)	>20:1 (71%)	1.8:1 (56%)
5	**3f**	**4f**	0.02	2.5 mol %	>20:1 (80%)	>20:1 (53%)	4.5:1 (65%)
6^c^	**3g**	**4g**	0.002	10.0 mol %	1.1:1 (36%)	2.6:1 (58%)	0.3:1 (14%)

^a^Reactions were run in toluene at 80 °C for 1 h, unless otherwise stated. ^b^The substrate to product ratio was determined from the ^1^H NMR-spectra of the crude reaction mixtures. ^c^Reaction was run in toluene at 110 °C for 3 h in the presence of 0.5 equiv of phenol.

### Ring closing enyne metathesis

**Scheme 3:**
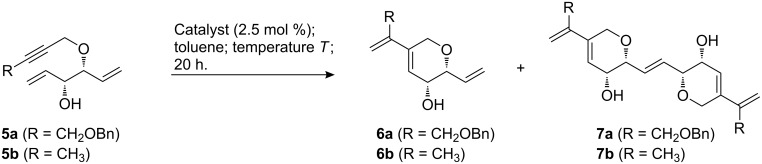
Ring closing enyne metathesis reactions.

Imahori et al. have recently discovered that allylic hydroxy groups significantly enhance the rate of ring closing enyne metathesis reactions [53–54]. In these cases, addition of an ethylene atmosphere [55] which is normally considered to be mandatory for good results, is not required. We have recently investigated the highly selective enyne metathesis of substrates such as **5a**,**b** to dihydropyrans **6a**,**b** in the presence of first generation catalysts **B** and **C** [56]. Notably, no dihydrofurans or other isomers were observed. Sometimes, the selectivity decreases when the more reactive second generation catalysts are used [57–58], and we were therefore interested to test NHC-ligated catalysts in this transformation (Scheme 3).

The results for ring closing enyne metathesis reactions with indenylidene catalysts **E** and **H** are summarized in Table 3. Enyne **5a** reacted to afford the expected dihydropyran **6a** with high conversion and high selectivity in toluene at 80 °C and at 110 °C in the presence of phosphine complex **E** (Table 3, entries 1 and 2). The dimerization product **7a** was not detected, and although the ^1^H NMR spectra of the crude reaction mixtures showed only signals of **6a** and trace amounts of the starting material **5a**, the isolated yields are mediocre which can be attributed to a significant loss of material during purification. Significantly lower conversions of 50% and 80% were observed with the pyridine complex **H** at 80 °C or 110 °C, respectively. Again, ^1^H NMR-spectra of the crude reaction mixtures showed only signals for **6a** and the starting material **5a** and not even trace amounts of a dimerization product **7a** (Table 3, entries 3 and 4) were detected. Different results were found for the methyl substituted enyne precursor **5b**. Remarkably, with both catalysts and both reaction temperatures apparently identical conversions and product distributions were observed: In all cases (Table 3, entries 5–8) the starting material was fully consumed and the ^1^H NMR-spectra of the crude reaction mixtures revealed only the presence of dihydropyran **6b** and its dimer **7b** in roughly a 1:1 ratio. This ratio is reflected nicely in the isolated yields, which were determined for one example (Table 3, entry 8). From these results, it can be concluded that benzyloxy-substituted enyne **5a** is significantly less reactive in enyne metathesis reactions which becomes more obvious if the pyridine complex **H** is used as a catalyst. For other examples, we have previously observed that benzyl ether moieties in close proximity to a C–C-multiple bond retard or inhibit metathesis reactions [50]. Presumably, partial catalyst deactivation by coordination of the benzyloxy group to the metal plays a role, and this might also explain why no dimerization product **7a** was observed, whereas **7b**, the self-metathesis product of **6b**, was isolated in significant quantities, which might suggest a remarkable residual activity of the catalysts after completion of the enyne metathesis.

**Table 3:** Ring closing enyne metathesis reactions catalyzed with **E** and **H**.

Entry	**5**	–R	Catalyst^a^	*T*	Conversion^b^	Ratio of **6**:**7**^b^	Product (Yield)

1	**5a**	–CH_2_OBn	**E**	80 °C	95%	>20:1	**6a** (50%)
2	**5a**	–CH_2_OBn	**E**	110 °C	95%	>20:1	**6a** (44%)
3	**5a**	–CH_2_OBn	**H**	80 °C	50%	>20:1	**6a** (29%) **5a** (21%)
4	**5a**	–CH_2_OBn	**H**	110 °C	80%	>20:1	**6a** (34%)
5	**5b**	–CH_3_	**E**	80 °C	100%	10:10.7	n. d.
6	**5b**	–CH_3_	**E**	110 °C	100%	10:10.3	n. d.
7	**5b**	–CH_3_	**H**	80 °C	100%	10:10.2	n. d.
8	**5b**	–CH_3_	**H**	110 °C	100%	10:9.6	**6b** (24%) **7b** (45%)

^a^2.5 mol % of catalyst, toluene as a solvent and an initial substrate concentration of 0.1 mol/L were used in all experiments. ^b^Rates of conversion and monomer/dimer ratios were determined from the ^1^H NMR-spectra of the crude reaction mixtures, which showed only signals of starting materials **5**, dihydropyrans **6** and dimers **7**.

The results discussed above for the less reactive enyne **5a** suggest that pyridine complex **H** is less active than **E** in enyne metathesis reactions, however, the results for the apparently more reactive substrate **5b** may indicate that the gap in catalytic activity between **E** and **H** is much smaller for ring closing enyne reactions than for ring closing acrylate metathesis reactions. However, the remarkably high amount of homodimerization product **7b** points to considerable activity of **H** in cross metathesis reactions, a peculiarity which has previously been noted for the bis(pyridine) complex **G** [33–35].

### Cross metathesis reactions

**Scheme 4:**
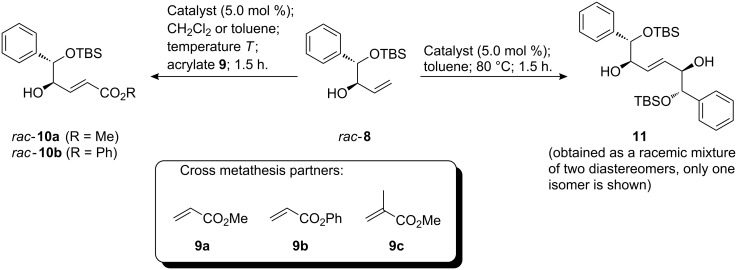
Cross metathesis reactions with allylic alcohol **8**.

Allylic alcohol **8** [59] was chosen to test the catalysts investigated in this study for cross metathesis activity, because allylic alcohols are known to undergo undesired “redox isomerization” in the presence of Ru metathesis catalysts in some cases with the formation of ethyl ketones [47,60]. Therefore, **8** can be considered as a rather challenging substrate. As partners in the cross metathesis reactions, three acrylates **9a**–**c** were chosen. In addition, homodimerization of **8** to diol **11** was also investigated (Scheme 4).

The results are summarized in Table 4. With methyl acrylate **9a** in dichloromethane at 40 °C, only the benzylidene catalyst **D** showed a satisfactory rate of conversion (Table 4, entry 1). A significantly lower conversion was observed under these conditions for pyridine complex **H** (Table 4, entry 3), however, the isolated yields were similar in both cases. Surprisingly, with catalyst **E** conversion was incomplete and a considerable amount of dimer **11** was formed (Table 4, entry 2). Compound **11** is an inseparable mixture of diastereomers, because **10a** was used as a racemate. Raising the temperature to 80 °C in toluene solved the problems of incomplete conversion and competing dimerization for all three catalysts (Table 4, entries 4–6) and *rac*-**10a** was isolated in comparable yields of between 83% and 86%. Phenyl acrylate (**9b**) is a less reactive cross metathesis partner, and all three catalysts led to incomplete conversion (Table 4, entries 7–9). The best result in this series was achieved with catalyst **H**, which gave a 2:1 ratio of product **10b** and starting material **8**, and an isolated yield of 60% (Table 4, entry 9). Methyl methacrylate (**9c**) did not react in cross metathesis reactions with **8** using either **D** or **H**. Instead, dimerization to compound **11** occurred. We [50] and others [61] have recently observed a considerable reduction of isomerization side reactions if acrylates are present during a metathesis reaction. Therefore, **8** was subjected to olefin metathesis conditions in the absence of acrylates to check if any isomerization occurred, or if dimer **11** was the preferred or sole product. With all three catalysts similar results were obtained: The starting material was rapidly and almost completely consumed, and the two diastereomers of **11** were the only detectable products in the reaction mixture (Table 4, entries 12–14).

**Table 4:** Cross metathesis reactions catalyzed by **D**, **E**, and **H**.

Entry	Acrylate	Solvent	*T*	Catalyst^a^	Ratio of **10**:**8**:**11**^b^	Product (Yield)^c^

1	**9a**	CH_2_Cl_2_	40 °C	**D**	16:1:0	*rac-***10a** (72%)
2	**9a**	CH_2_Cl_2_	40 °C	**E**	1.4:1.3:1	n. d.
3	**9a**	CH_2_Cl_2_	40 °C	**H**	3.7:1:0	*rac*-**10a** (65%)
4	**9a**	toluene	80 °C	**D**	1:0:0	*rac*-**10a** (84%)
5	**9a**	toluene	80 °C	**E**	1:0:0	*rac*-**10a** (83%)
6	**9a**	toluene	80 °C	**H**	1:0:0	*rac*-**10a** (86%)
7	**9b**	toluene	80 °C	**D**	1.2:1:0	*rac*-**10b** (53%)
8	**9b**	toluene	80 °C	**E**	1:1:0	*rac*-**10b** (47%)
9	**9b**	toluene	80 °C	**H**	2:1:0	*rac*-**10b** (60%)
10	**9c**	toluene	80 °C	**D**	0:0:1	**11** (n. d.)
11	**9c**	toluene	80 °C	**H**	0:0:1	**11** (n. d.)
12	—	toluene	80 °C	**D**	—:1:30	**11** (68%)
13	—	toluene	80 °C	**E**	—:1:9	**11** (n. d.)
14	—	toluene	80 °C	**H**	—:1:16	**11** (n. d.)

^a^A catalyst loading of 5.0 mol % was used in all experiments. ^b^Ratios were determined from the ^1^H NMR-spectra of crude reaction mixtures. ^c^All cross metathesis products were obtained as single *E*-isomers.

These results demonstrate that the novel catalyst **H**, albeit significantly less active than **D** or **E** in the ring closing metathesis of acrylates, appears to be competitive in cross metathesis reactions.

## Conclusion

In conclusion, we have evaluated and compared the catalytic performance of two indenylidene NHC-metathesis catalysts and the well established second generation Grubbs catalyst in various small molecule metathesis reactions. The activity of the mixed NHC-phosphine catalysts **D** and **E** appears to be similar in most applications. Some results hint at a somewhat faster reaction with benzylidene complex **D**, while **E** apparently performs slightly better in “slow” metathesis reactions, presumably since it is more robust. The novel pyridine complex **H** was also tested in several olefin metathesis reactions. While this catalyst gives rather unsatisfactory results in the ring closing metathesis of acrylates, its performance in ring closing enyne metathesis reactions is only slightly lower than the phosphine complexes **D** and **E**. However, the activity of **H** in cross metathesis reactions is similar to **D** and **E**. Furthermore, initial studies concerning the use of unconventional solvents revealed that **H** might be quite active, at least for some applications, in acetic acid.

## Experimental

All experiments were conducted in dry reaction vessels under an atmosphere of dry argon. Solvents were purified by standard procedures. All yields and conversions reported herein are average values of at least two experiments. ^1^H NMR spectra were obtained at 300 MHz in CDCl_3_ with CHCl_3_ (δ = 7.26 ppm) as the internal standard. Coupling constants (*J*) are given in Hz. ^13^C NMR spectra were recorded at 75 MHz in CDCl_3_ with CDCl_3_ (δ = 77.0 ppm) as the internal standard. IR spectra were recorded as films on NaCl or KBr plates or as KBr discs. Wavenumbers (ν) are given in cm^–1^. Mass spectra were obtained at 70 eV. Whenever known compounds were used as starting materials, reagents or catalysts, they were either purchased or were synthesized following literature procedures: **1** [62], **3a** [63], **3b**–**3e** [50], **3g** [51], **5a**,**b** [56]. Catalyst **D** was purchased from Aldrich and used without further purification. Catalysts **E** and **H** were donated by Umicore, Hanau, Germany, and also used without further purification. The following products have previously been synthesized via olefin metathesis reactions under different conditions: **2** [62], **4a** [63], **4b**–**4e** [50], **4f** [64], **4g** [51], **6a**,**b** [56].

### General procedure for the RCM of 1: variation of solvent, catalyst and catalyst loading

Allyl ether **1** (94.0 mg, 0.5 mmol) was dissolved in the appropriate dry and degassed solvent (5.0 mL). Catalyst **D** (4.2 mg for 1.0 mol %), **E** (4.7 mg for 1.0 mol %) or **H** (3.7 mg for 1.0 mol %, 7.4 mg for 2.0 mol %, 11.2 mg for 3.0 mol %, 15.0 mg for 4.0 mol % or 18.8 mg for 5.0 mol %) was then added. Immediately after addition of the catalyst, the reaction vessel was immersed in an oil bath preheated to 40 °C (electronic temperature control) for a period of time between 60 and 62 min. After this time, the reaction vessel was allowed to cool to ambient temperature, the solvent was removed by evaporation, and the residue immediately subjected to NMR spectroscopy. The ratio of dihydropyran **2** to allyl ether **1** was determined by integration of characteristic, baseline separated signals. Each experiment was repeated at least two times. The reported rates of conversion are average values.

#### Ring closing metathesis of acrylates

**General procedure for the synthesis of furanones 4b–4f by RCM:** To a solution of the appropriate acrylate **3** (1.0 mmol) in dry and degassed toluene (50 mL for 0.02 mol·L^–1^ or 100 mL for 0.01 mol·L^–1^), was added either catalyst **D** (21.2 mg for 2.5 mol % or 42.4 mg for 5.0 mol %), **E** (23.7 mg for 2.5 mol % or 47.4 mg for 5.0 mol %) or **H** (18.7 mg for 2.5 mol % or 37.4 mg for 5.0 mol %). The solution was heated to 80 °C for 90 min. The solvent was then removed by evaporation and the residue purified by flash column chromatography on silica to give the corresponding lactones **4**. The ratio of lactone **4** to acrylate **3** was determined by integration of characteristic, baseline separated signals in the ^1^H NMR-spectrum of the crude reaction mixture. Representative example: **5-phenylfuran-2(5*H*)-one (4f)**. This compound was obtained as a colourless oil from **3f** (189 mg, 1.0 mmol) following the general procedure. Yield of **4f** using catalyst **D**: 128 mg (0.80 mmol, 80%). Yield of **4f** using catalyst **E**: 85 mg (0.53 mmol, 53%). Yield of **4f** using catalyst **H**: 104 mg (0.65 mmol, 65%). ^1^H NMR (300 MHz, CDCl_3_) δ 7.53 (dd, *J* = 5.7, 1.7, 1H), 7.45–7.35 (3H), 7.30–7.23 (2H), 6.23 (dd, *J* = 5.7, 2.1, 1H), 6.01 (t (br), *J* = 1.9, 1H); ^13^C NMR (75 MHz, CDCl_3_) δ 173.0 (0), 155.8 (1), 134.4 (0), 129.3 (1), 129.0 (1), 126.5 (1), 121.0 (1), 84.3 (1); HRMS (ESI) calcd for C_10_H_8_O_2_Na [M+Na]^+^: 183.0422, found: 183.0439.

**Procedure for the synthesis of 6-phenyl-5,6-dihydropyran-2-one (4a):** The acrylate **3a** (201 mg, 1.0 mmol) was dissolved in dry and degassed toluene (100 mL). After adding the catalyst (**D**: 42.4 mg **B**: 47.4 mg **H**: 37.4 mg, 0.05 mmol, 5 mol %) the solution was stirred for 3 h at 80 °C. After cooling the solution to ambient temperature, all volatiles were evaporated. The residue was purified by chromatography on silica (hexane/MTBE 2:1). Yield of **4a** using catalyst **D**: 139 mg (0.80 mmol, 80%). Yield of **4a** using catalyst **E**: 160 mg (0.92 mmol, 92%). Yield of **4a** using catalyst **H**: 170 mg (0.41 mmol, 41%). The ratios of lactone **4a** to acrylate **3a** were determined by integration of characteristic, baseline separated signals in the ^1^H NMR-spectrum of the crude reaction mixture. mp 58–59 °C; ^1^H NMR (300 MHz, CDCl_3_) δ 7.43–7.33 (5H), 6.97 (ddd, *J* = 9.7, 5.7, 2.6, 1H), 6.13 (ddd, *J* = 9.7, 1.1, 1.1, 1H), 5.45 (dd, *J* = 11.2, 4.8, 1H), 2.70–2.57 (2H); ^13^C NMR (75 MHz, CDCl_3_) δ 164.0 (0), 144.8 (1), 138.4 (0), 128.6 (1), 128.6 (1), 126.0 (1), 121.7 (1), 79.2 (1), 31.6 (2); IR (neat) ν 3064 (w), 2904 (w), 1716 (s), 1381 (m), 1242 (s), 1059 (m), 1020 (m), 909 (m); EIMS (%) *m/z* 174 (M^+^, 6), 77 (13), 68 (100), 39 (63); HRMS (EI) calcd. for C_11_H_10_O_2_ [M^+^]: 174.0675, found 174.0689; Anal. calcd for C_11_H_10_O_2_: C, 75.8, H, 5.8; found: C, 75.6, H, 5.8.

**Procedure for the synthesis of (*R*)-2-((2*R*,3*R*)-3-(*tert*-butyldimethylsilyloxy)-6-oxo-3,6-dihydro-2*H*-pyran-2-yl)-2-hydroxyethyl benzoate (4g):** The acrylate **3g** (330 mg, 0.78 mmol) and phenol (37 mg, 0.39 mmol) were dissolved in dry and degassed toluene (390 mL). After adding the catalyst (**D**: 66.1 mg **B**: 73.9 mg **H**: 58.3 mg, 0.078 mmol, 10 mol %), the solution was stirred for 3 h at 80 °C. The solution was cooled to ambient temperature and all volatiles were evaporated. The residue was purified by chromatography on silica. Yield of **4g** using catalyst **D**: 110 mg (0.28 mmol, 36%). Yield of **4g** using catalyst **E**: 177 mg (0.45 mmol, 58%). Yield of **4g** using catalyst **H**: 43 mg (0.11 mmol, 14%). The ratios of lactone **4g** to acrylate **3g** were determined by integration of characteristic, baseline separated signals in the ^1^H NMR-spectrum of the crude reaction mixture. All analytical data were identical to those reported previously in the literature [51].

#### Ring closing enyne metathesis reactions

**General procedure:** To a solution of the corresponding precursor **5** (2.0 mmol) in toluene (40 mL), was added catalyst **E** (47.4 mg, 2.5 mol %) or **H** (37.4 mg, 2.5 mol %). The solution was heated to the appropriate temperature (80 °C or 110 °C) for 20 h, then cooled to ambient temperature and the solvent evaporated. The crude product was analyzed by ^1^H and ^13^C NMR spectroscopy. Analytically pure samples were obtained by column chromatography on silica. Representative example: **Ring closing enyne metathesis of 5b.** Following the general procedure, **5b** (330 mg, 2.0 mmol) was treated with catalyst **H** (37.4 mg, 2.5 mol %) at 80 °C. After column chromatography, **6b** (80 mg, 0.48 mmol, 24%) and **7b (**274 mg, 0.90 mmol, 45%) were isolated. The rate of conversion and the ratios of **6b** to **7b** were determined by integration of characteristic, baseline separated signals in the ^1^H NMR-spectrum of the crude reaction mixture. All analytical data of **6b** were identical to those reported previously in the literature.[56] Analytical data for **(2*R*,2'*R*,3*R*,3'*R*,*E*)-2,2'-(ethene-1,2-diyl)bis(5-(prop-1-en-2-yl)-3,6-dihydro-2*H*-pyran-3-ol (7b):** [α]_D_^25^: –390.0 (*c* 0.13, CH_2_Cl_2_); ^1^H NMR (300 MHz, CDCl) δ 6.05 (d, *J* = 5.4, 2H); 5.96 (d, *J* = 1.7, 2H); 4.93 (s, 1H); 4.83 (s, 2H); 4.49 (d, *J* = 15.6, 2H); 4.25 (d, *J* = 15.5, 2H); 4.04 (s, 2H); 3.99 (d, *J* = 5.5, 1H); 3.32 (s, 2H); 1.87 (s, 6H); ^13^C NMR (75 MHz, CDCl_3_) δ 139.5 (0), 138.5 (0); 129.1 (2), 122.8 (1); 112.3, (2); 77.3 (1), 66.2 (2); 64.2 (1); 20.1 (3); IR ν 3297 (s); 3087 (m); 2945 (m); 2828 (m); 1606 (m); 1371 (w); 1124 (s); 1103 (m); 1055 (m), 882 (s), 827 (s); HRMS (EI) calcd for C_18_H_24_O_4_ [M]^+^: 304.1675, found: 304.1683; Anal. calcd for C_18_H_24_O_4_: C, 71.0, H, 8.0, found: C, 70.8, H, 7.7.

#### Cross-metathesis reactions

**General procedure:** A solution of the allylic alcohol *rac-***8** (139 mg, 0.5 mmol) and the corresponding acrylate **9** (5 mmol) in dry and degassed toluene (1.0 mL for 0.5 mol·L^–1^, 5.0 mL for 0.1 mol·L^–1^) was warmed to approximately 45 °C. Then the catalyst (0.025 mmol, 5 mol %, **D**: 42.4 mg **E**: 47.4 mg **H**: 18.7 mg) was added to the solution and stirred for 90 min at 80 °C. After cooling to ambient temperature all volatiles were evaporated and the residue purified by chromatography on silica. The rates of conversion and the ratios of **10** to **8** to **11** were determined by integration of characteristic, baseline separated signals in the ^1^H NMR-spectrum of the crude reaction mixture.

**(4*RS*,5*SR*,*E*)-methyl 5-(*tert*-butyldimethylsilyloxy)-4-hydroxy-5-phenylpent-2-enoate (*rac*-10a).** Obtained as colourless oil from *rac-***8** (139 mg, 0.5 mmol) and methyl acrylate (**9a**) following the general procedure. Yield of *rac-***10a** using catalyst **D**: 141 mg (0.42 mmol, 84%). Yield of *rac-***10a** using catalyst **E**: 139 mg (0.41 mmol, 83%). Yield of *rac-***10a** using catalyst **H**: 145 mg (0.43 mmol, 86%). ^1^H NMR (300 MHz, CDCl_3_) δ 7.37–7.25 (5 H), 6.73 (dd, *J* = 15.7, 4.1, 1 H), 6.07 (dd, *J* = 15.7, 1.9, 1 H), 4.47 (d, *J* = 6.8, 1 H), 4.29 (dddd, *J* = 6.6, 4.1, 3.6, 2.0, 1 H), 3.70 (s, 3 H), 2.90 (d, *J* = 3.9, 1 H), 0.89 (s, 9 H), 0.03 (s, 3 H), −0.18 (s, 3 H); ^13^C NMR (75 MHz, CDCl_3_) δ 166.7 (0), 145.8 (1), 140.2 (0), 128.4 (1), 128.2 (1), 126.9 (1), 121.6 (1), 78.4 (1), 75.8 (1), 51.5 (3), 25.7 (3), 18.1 (0), −4.6 (3), −5.1 (3); IR ν 3492 (w), 2952 (w), 2857 (w), 1724 (m), 1253 (m), 1068 (m), 835 (s), 777 (s); HRMS (ESI) calcd for C_18_H_28_O_4_NaSi [M+Na]^+^: 359.1655, found: 359.1652; EIMS (%) *m/z* = 337 (M^+^, 1), 221 (100), 187 (11), 173 (6), 145 (9), 115 (11), 91 (6), 73 (15); Anal. calcld C_18_H_28_O_4_:C, 64.3, H: 8.4; found: C: 64.2, H: 8.4.

**(4*RS*,5*SR*,*E*)-phenyl 5-(*tert*-butyldimethylsilyloxy)-4-hydroxy-5-phenylpent-2-enoate (*rac*-10b).** Obtained as colourless solid from *rac-***8** (139 mg, 0.5 mmol) and phenyl acrylate (**9b**) following the general procedure. Yield of *rac-***10b** using catalyst **D**: 105 mg (0.27 mmol, 53%). Yield of *rac-***10a** using catalyst **E**: 94 mg (0.30 mmol, 47%). Yield of *rac-***10a** using catalyst **H**: 119 mg (0.43 mmol, 60%). mp 82–84 °C; ^1^H NMR (300 MHz, CDCl_3_) δ 7.41–7.18 (8 H), 7.12–7.06 (2 H), 6.93 (dd, *J* = 15.6, 4.0, 1 H), 6.30 (dd, *J* = 15.6, 1.8, 1 H), 4.55 (d, *J* = 6.6, 1 H), 4.37 (dddd, *J* = 6.1, 4.2, 4.0, 1.9, 1 H), 2.96 (d, *J* = 4.0, 1 H), 0.91 (s, 9 H), 0.06 (s, 3 H), −0.16 (s, 3 H); ^13^C NMR (75 MHz, CDCl_3_) δ 164.6 (0), 150.7 (0), 147.9 (1), 140.2 (0), 129.3 (1), 128.5 (1), 128.3 (1), 126.9 (1), 125.7 (1), 121.5 (1), 121.3 (1), 78.4 (1), 76.0 (1), 25.8 (3), 18.2 (0), −4.5 (3), −5.1 (3); IR ν 3545 (w), 2929 (w), 2857 (w), 1739 (s), 1493 (m), 1194 (s), 836 (s), 778 (s); HRMS (ESI) calcd for C_23_H_30_O_4_NaSi [M+Na]^+^: 421.1811, found: 421.1800; EIMS (%) *m/z* = 341 (2), 332 (2), 292 (1), 249 (3), 247 (4), 221 (100), 75 (21) 73 (47); Anal. calcd C, 69.3, H, 7.6; found C, 69.4, H, 7.5.

**Dimerization of *rac*-8 (Compound 11).** The catalyst (5 mol %, 0.029 mmol, **D**: 24.2 mg, **E**: 27.0 mg, **H**: 21.3 mg) was added to a solution of alcohol *rac-***8** (150 mg, 0.57 mmol) in dry and degassed toluene (1.13 mL, 0.5 M). The reaction mixture was stirred for 90 min at 80 °C. After cooling to room temperature all volatiles were evaporated. The crude product **11** was purified by chromatography on silica. Compound **11** was isolated as a partially separable mixture of diastereomers (combined yield for catalyst **D**: 107 mg, 0.39 mmol, 68%). The rates of conversion were determined by integration of characteristic, baseline separated signals in the ^1^H NMR-spectrum of the crude reaction mixture. Analytical data for diastereomer **11a**: ^1^H NMR (300 MHz, CDCl_3_) δ 7.30–7.24 (3H), 7.11–7.06 (2H), 5.44 (dd, *J* = 2.6, 0.9, 1H), 4.20 (d, *J* = 7.5, 1H), 4.00 (dm, *J* = 7.4, 1 H), 2.83 (d, *J* = 2.4, 1H), 0.87 (s, 9H), 0.01 (s, 3H), −0.22 (s, 3H); ^13^C NMR (75 MHz, CDCl_3_) δ 141.0 (0), 129.9 (1), 128.0 (1), 127.7 (1), 127.3 (1), 79.5 (1), 76.7 (1), 25.8 (3), 18.1 (0), −4.5 (3), −5.1 (3); HRMS (ESI) calcd for C_30_H_48_O_4_Si_2_Na [M+Na]^+^: 551.2989, found: 551.3010; EIMS (%) *m*/*z* = 283 (4), 189 (4), 157 (56), 129 (16), 103 (22), 99 (100). Analytical data for diastereomer **11b**: ^1^H NMR (300 MHz, CDCl_3_) δ 7.33–7.17 (5H), 5.52 (dd, *J* = 2.6, 0.9, 1H), 4.38 (d, *J* = 6.6, 1 H), 4.03 (ddm, *J* = 6.0, 3.0, 1H), 2.74 (d, *J* = 3.4, 1H), 0.89 (s, 9H), 0.03 (s, 3H), −0.19 (s, 3H); ^13^C NMR (75 MHz, CDCl_3_) δ 141.0 (0), 130.3 (1), 128.0 (1), 127.7 (1), 127.1 (1), 79.0 (1), 76.4 (1), 25.8 (3), 18.1 (0), −4.6 (3), −5.1 (3).

## Supporting Information

File 1 Experimental procedures and characterization data for starting materials which have previously not been described in the literature; representative illustrations for determination of conversions; copies of spectra for new compounds.
