# Supplementary material for: The catalytic performance of Ru–NHC alkylidene complexes: PCy3 versus pyridine as the dissociating ligand
Source: Beilstein J Org Chem. 2010 Dec 15;6:1188–98. doi: 10.3762/bjoc.6.136 (PMC3028929; doi:10.3762/bjoc.6.136)
Supplement: File 1 — Experimental procedures and characterization data for starting materials which have previously not been described in the literature; representative illustrations for determination of conversions; copies of spectra for new compounds. [file Beilstein_J_Org_Chem-06-1188-s001.pdf]

Supporting Information
for

The catalytic performance of Ru–NHC alkylidene complexes: PCy_3 versus pyridine as the dissociating ligand

Stefan Krehl, Diana Geißler, Sylvia Hauke, Oliver Kunz, Lucia Staude and Bernd Schmidt*

Address: Universität Potsdam, Institut für Chemie, Organische Synthesechemie, Karl-Liebknecht-Straße 24-25, D-14476 Golm, Germany.

Email: Bernd Schmidt - bernd.schmidt@uni-potsdam.de
* Corresponding author

Table of Contents:

A Synthesis of starting materials **3a**, **3f**, *rac*-**8**

B Determination of rates of conversion for representative examples and procedure for isolation of compound **2** from RCM in acetic acid

C Copies of spectra for compounds **3a**, **3f**, **4a**, **4f**, **7b**, *rac*-**8**, *rac*-**8'**, *rac*-**8''**, *rac*-**10a**, *rac*-**10b**, **11a**, **11b**

A Synthesis of starting materials 3a, 3f, *rac*-8

1-Phenylbut-3-enyl acrylate (3a).

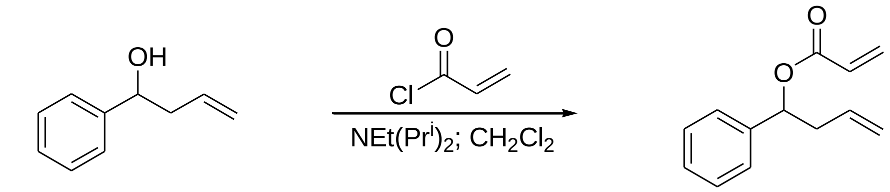

1-Phenylbut-3-en-1-ol **3a**

To a solution of 1-phenylbut-3-en-1-ol (2.10 g, 14.2 mmol) in dry degassed CH_2Cl_2 (280 mL), was added *N*-ethyldiisopropylamine (40.5 mmol, 1.7 mL). The solution was cooled to 0 °C, and a solution of acryloyl chloride (33.8 mmol, 2.80 mL) in dry degassed CH_2Cl_2 (90 mL) slowly added. The mixture was warmed to ambient temperature, poured into water, and the organic layer separated. The aqueous layer was extracted with CH_2Cl_2, and the combined organic extracts were dried with $MgSO_4$, filtered, and all the volatiles were evaporated. The residue was purified by chromatography on silica to give **3a** (2.7 g, 13.5 mmol, 95%) as a colourless liquid. ^{1}H NMR (300 MHz, $CDCl_3$) δ 7.39-7.27 (5 H), 6.43 (dd, *J* = 17.4, 1.4, 1 H), 6.16 (dd, *J* = 17.3, 10.4, 1 H), 5.89 (dd, *J* = 7.4, 6.2, 1 H), 5.83 (dd, *J* = 10.4, 1.4, 1 H), 5.72 (dddd, *J* = 17.1, 10.2, 7.1, 6.9, 1 H), 5.09 (dd, *J* = 17.1, 1.4, 1 H), 5.06 (dm, *J* = 10.1, 1 H), 2.72 (dddm, *J* = 14.3, 7.3, 7.1, 1 H), 2.75 (dddm, *J* = 14.1, 7.4, 6.8, 1.4, 1 H); ^{13}C NMR (75 MHz, $CDCl_3$) δ 165.3 (0), 140.0 (0), 133.2 (1), 130.7 (2), 128.6 (1), 128.4 (1), 127.9 (1), 126.5 (1), 118.0 (2), 75.3 (1), 40.7 (2); HRMS (ESI) calcd for $C_{13}H_{14}O_2Na$ $[M+Na]^+$: 225.0891, found: 225.0906; EIMS (%) *m/z* = 202 (M^+, 1), 161 (37), 129 (26), 86 (31), 84 (55), 55 (100), 49 (75), 36 (46).

1-Phenylallyl acrylate (3f).

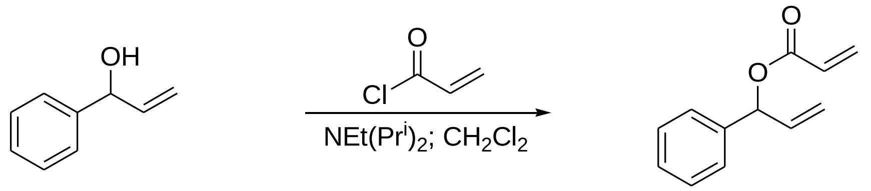

1-phenylprop-2-en-1-ol **3f**

Following the procedure given above for **3a**, the title compound was obtained from 1-phenylprop-2-en-1-ol (2.0 g, 14.9 mmol). Yield of **3f**: 2.20 g (11.6 mmol, 78%). ^{1}H NMR (300 MHz, $CDCl_3$) δ 7.42–7.26 (5 H), 6.46 (dd, *J* = 17.3, 1.4, 1 H), 6.35 (d, *J* = 5.9 1 H), 6.19 (dd, *J* = 17.3, 10.4, 1 H), 6.05 (ddd, *J* = 17.0, 10.4, 5.9, 1 H), 5.86 (dd, *J* = 10.4, 1.4, 1 H), 5.31 (d, *J* = 17.2, 1.4, 1 H), 5.27 (d, *J* = 10.4, 1 H); ^{13}C NMR (75 MHz, $CDCl_3$) δ 165.1 (0), 138.8 (0), 136.2 (1), 131.1 (1), 128.6 (1), 128.5 (2), 128.2 (1), 127.1 (1), 117.0 (2), 76.3 (1), IR (neat) ν 3034 (w), 1722 (s), 1634 (w), 1403 (m), 1260 s), 1175 (s); HRMS (ESI) calcd

for $C_{12}H_{12}O_2$ $[M]^+$: 188.0832, found: 188.0844; Anal. calcd for $C_{12}H_{12}O_2$: C, 76.6, H, 6.4; found: C, 76.7, H, 6.3.

(1*SR*, 2*RS*)-1-(*tert*-butyldimethylsilyloxy)-1-phenylbut-3-en-2-ol (*rac*-8) [1].

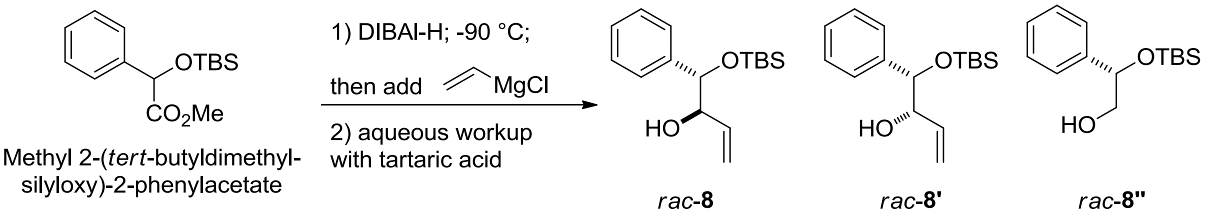

A solution of methyl-2-(*tert*-butyldimethylsilyloxy-2-phenylacetate [2] (3.00 g, 10.7 mmol) in dry dichloromethane (50 mL) was cooled to –90 °C. DIBAL-H (11.7 mL, 12.8 mmol, 1.1 m in cyclohexane) was added slowly. After complete consumption of the starting material (monitored by TLC, approximately 15 min) a solution of vinylmagnesium chloride (9.4 mL, 16.1 mmol, 1.7 m in THF) was added. The solution was allowed to warm to ambient temperature over 12 h. The reaction mixture was then poured into a mixture of water and MTBE (1:1, 100 mL). After adding a saturated aqueous solution of tartaric acid (50 mL) the aqueous phase was extracted three times with MTBE. The combined organic extracts were dried with $MgSO_4$, filtered and evaporated. Chromatography on silica gave *rac*-**8** (960 mg, 3.6 mmol, 34%), *rac*-**8'** (731 mg, 2.8 mmol, 26%) and *rac*-**8''** (821 mg, 3.4 mmol, 32%). Cross metathesis experiments were conducted with rac-8 as a single diastereomer.

Analytical data for (1*SR*,2*RS*)-1-(*tert*-butyldimethylsilyloxy)-1-phenylbut-3-en-2-ol (*rac*-**8**): ^{1}H NMR (300 MHz, $CDCl_3$) δ 7.36–7.23 (5 H), 5.67 (ddd, *J* = 17.2, 10.6, 5.4, 1 H), 5.20 (ddd, *J* = 17.3, 1.7, 1.6, 1 H), 5.10 (ddd, *J* = 10.6, 1.6, 1.5, 1 H), 4.43 (d, *J* = 6.8, 1 H), 4.11 (ddddd, *J* = 6.7, 5.3, 3.6, 1.8, 1.4, 1 H), 2.82 (d, *J* = 3.6, 1 H), 0.90 (s, 9 H), 0.05 (s, 3 H), −0.20 (s, 3 H); ^{13}C NMR (75 MHz, $CDCl_3$) δ 140.9 (0), 136.2 (1), 128.0 (1), 127.8 (1), 127.2 (1), 116.5 (2), 79.1 (1), 77.4 (1), 25.8 (3), 18.2 (0), −4.5 (3), −5.1 (3); IR (neat) ν 2929 (w), 2857 (w), 1252 (m), 1069 (m), 834 s), 777 (m); HRMS (ESI) calcld for $C_{16}H_{26}O_2NaSi$ $[M+Na]^+$: 301.1600, found: 301.1626; EIMS (%) *m/z* = 279 (M^+, 1), 221 (100), 203 (10), 163 (20), 149 (33), 129 (56), 115 (25), 91 (12), 73 (100), 57 (28); Anal. calcld for $C_{16}H_{26}O_2Si$: C, 69.0, H, 9.4; found: C: 68.9, H: 9.9.

Analytical data for (1*SR*,2*SR*)-1-(*tert*-butyldimethylsilyloxy)-1-phenylbut-3-en-2-ol (*rac*-**8'**): ^{1}H NMR (300 MHz, $CDCl_3$) δ 7.34–7.23 (5 H), 5.80 (ddd, *J* = 17.0, 10.5, 6.1, 1 H), 5.22 (ddd, *J* = 17.3, 1.5, 1.5, 1 H), 5.14 (ddd, *J* = 10.5, 1.5, 1.4, 1 H), 4.65 (d, *J* = 5.1, 1 H), 4.17 (dddd, *J* = 6.3, 5.1, 1.3, 1.2, 1 H), 2.05 (d, *J* = 5.1, 1 H), 0.90 (s, 9 H), 0.06 (s, 3 H), −0.14 (s, 3 H); ^{13}C NMR (75 MHz, $CDCl_3$) δ 140.8 (0), 136.6 (1), 128.0 (1), 127.6 (1), 127.1 (1),

116.7 (2), 78.1 (1), 77.3 (1), 25.8 (3), 18.2 (0), −4.6 (3), −5.0 (3); IR ν 3436 (w), 2929 (w), 2857 (w), 1252 (m), 1094 (m), 835 (s), 776 (m); HRMS (ESI) calcld for $C_{16}H_{26}O_2NaSi$ $[M+Na]^+$: 301.1600, found: 301.1627; EIMS (%) *m/z* = 279 (M^+, 1), 261 (3), 222 (20), 221 (100), 129 (30), 75 (42), 73 (56), 91 (12), 73 (100), 57 (28); Anal. calcld for $C_{16}H_{26}O_2Si$: C, 69.0, H, 9.4; found C, 68.7, H, 9.6.

Analytical data for *rac*-2-(*tert*-butyldimethylsilyloxy)-2-phenylethanol (*rac*-**8''**) [3]: ^{1}H NMR (300 MHz, $CDCl_3$) δ 7.35–7.25 (5 H), 4.77 (dd, *J* = 7.0, 4.7, 1 H), 3.60 (dd, *J* = 8.0, 4.6, 1 H), 3.58 (dd, *J* = 6.9, 5.1, 1 H), 2.08 (dd, *J* = 7.7, 5.3, 1 H), 0.92 (s, 9 H), 0.07 (s, 3 H), −0.09 (s, 3 H); ^{13}C NMR (75 MHz, $CDCl_3$) δ 141.4 (0), 128.2 (1), 127.7 (1), 126.3 (1), 75.9 (1), 69.0 (2), 25.8 (3), 18.2 (0), − 4.6 (3), − 5.0 (3). HRMS (ESI) calcld for $C_{14}H_{24}O_2NaSi$ $[M+Na]^+$: 275.1443, found: 275.1459.

B Determination of rates of conversion for representative examples and procedure for isolation of compound 2 from RCM in acetic acid

Figure S1: A typical ^{1}H NMR spectrum of a crude reaction mixture for RCM of allyl ether **1**.

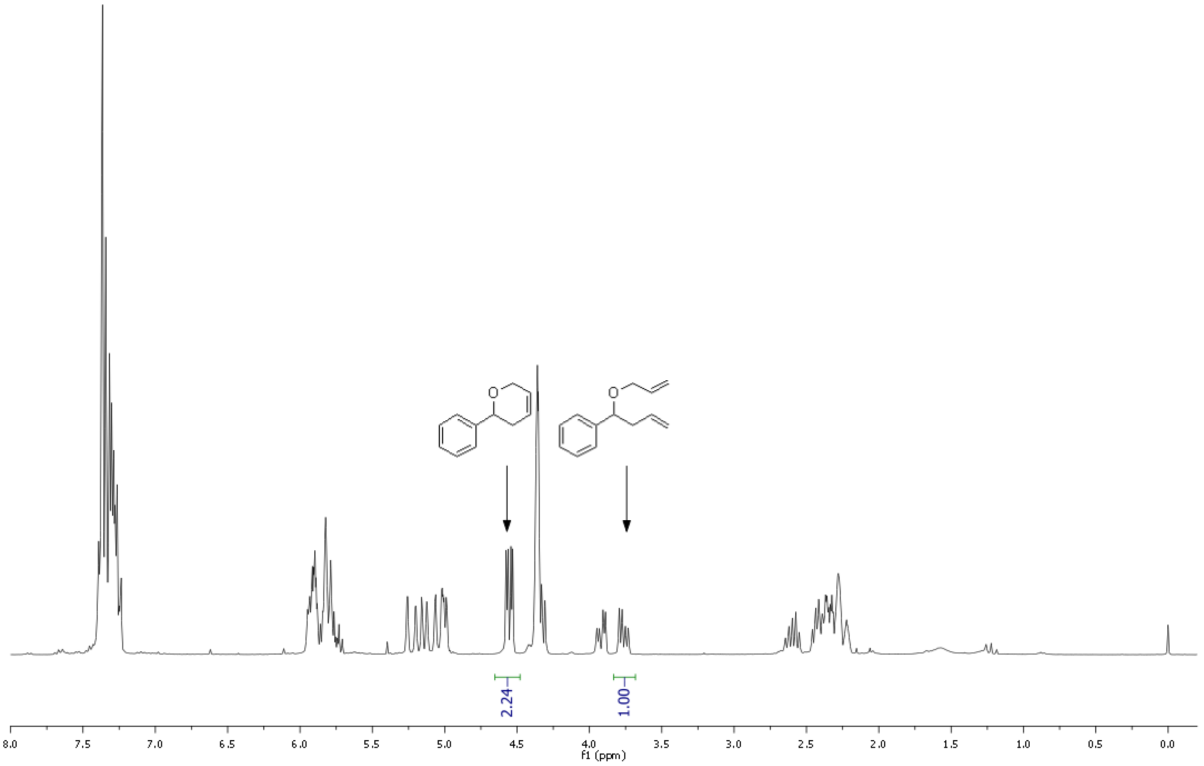

The example shown here is the ^{1}H NMR-spectrum of the crude reaction mixture obtained from the reaction of **1** in the presence of 1 mol % of catalyst **H** in acetic acid (refer to Figure 3 of main manuscript).

Ratios of product to starting material were determined from the integrals for –C*H*Ph- of compound **2** and –CH*H*O- for compound **1**. The same signals were used for the determination of product to starting material ratios in all experiments of one series.

Procedure for the ring closing metathesis of allyl ether 1 in acetic acid on preparative scale. Allyl ether **1** (94.0 mg, 0.5 mmol) was dissolved in acetic acid (used as purchased without further purification, 5 mL). Catalyst **H** (7.4 mg, 2.0 mol %) was then added. Immediately after addition of the catalyst, the reaction vessel was immersed in an oil bath preheated to 40 °C (electronic temperature control) for 60 min. After this time, the reaction vessel was allowed to cool to ambient temperature and the solvent removed by evaporation. The residue was purified by chromatography on silica to give **2** (64 mg, 80%).

Figure S2: A typical ^{1}H NMR spectrum of a crude reaction mixture for RCM of acrylate **3a**.

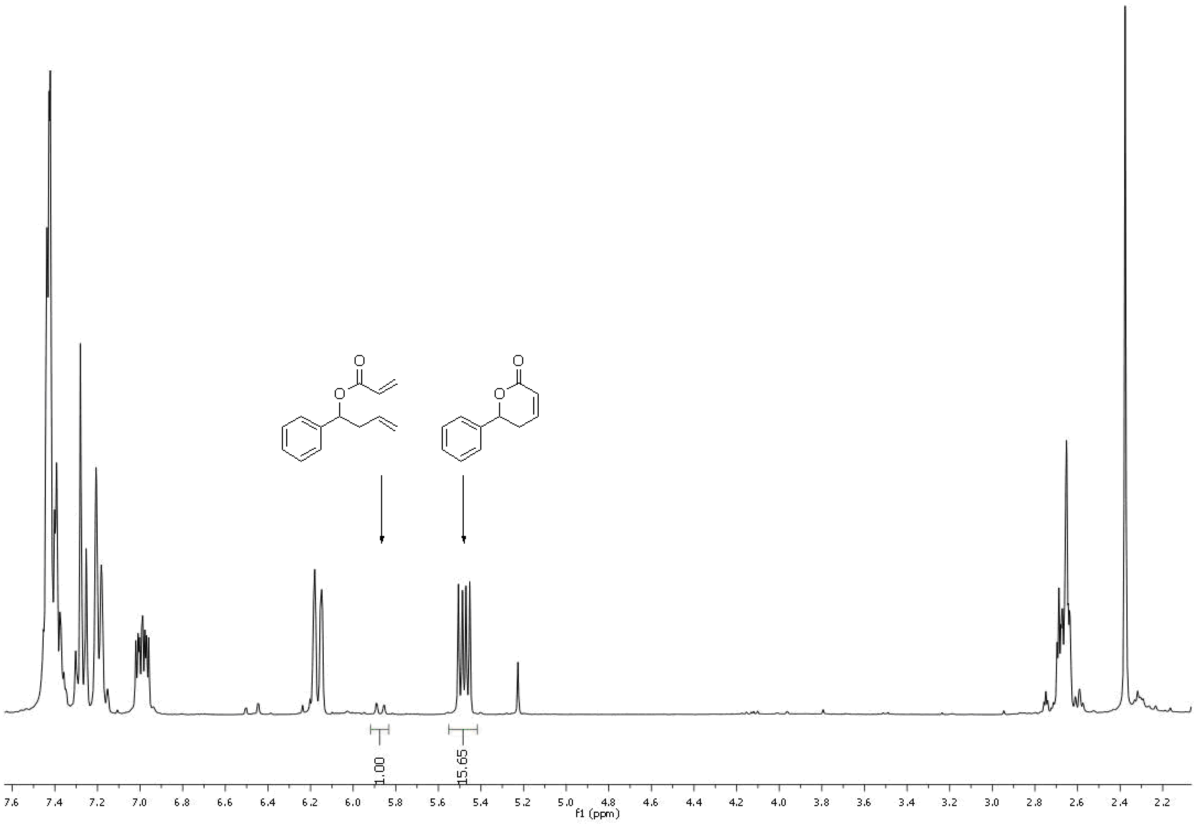

The example shown here is the ^{1}H NMR-spectrum of the crude reaction mixture obtained from the reaction of **3a** in the presence of 5 mol % of catalyst **D** in toluene (refer to Table 1, entry 1 of the main manuscript).

Ratios of product to starting material were determined from the integrals for –C*H*Ph- of compound **4a** and =CHH_{cis} for compound **3a**. The same signals were used for the determination of product to starting material ratios in all experiments of one series.

C Copies of spectra for compounds 3a, 3f, 4a, 4f, 7b, *rac*-8, *rac*-8’, *rac*-8’’, *rac*-10a, *rac*-10b, 11a, 11b

Figure S3: ^{1}H NMR spectrum of compound **3a** ($CDCl_3$, 300 MHz).

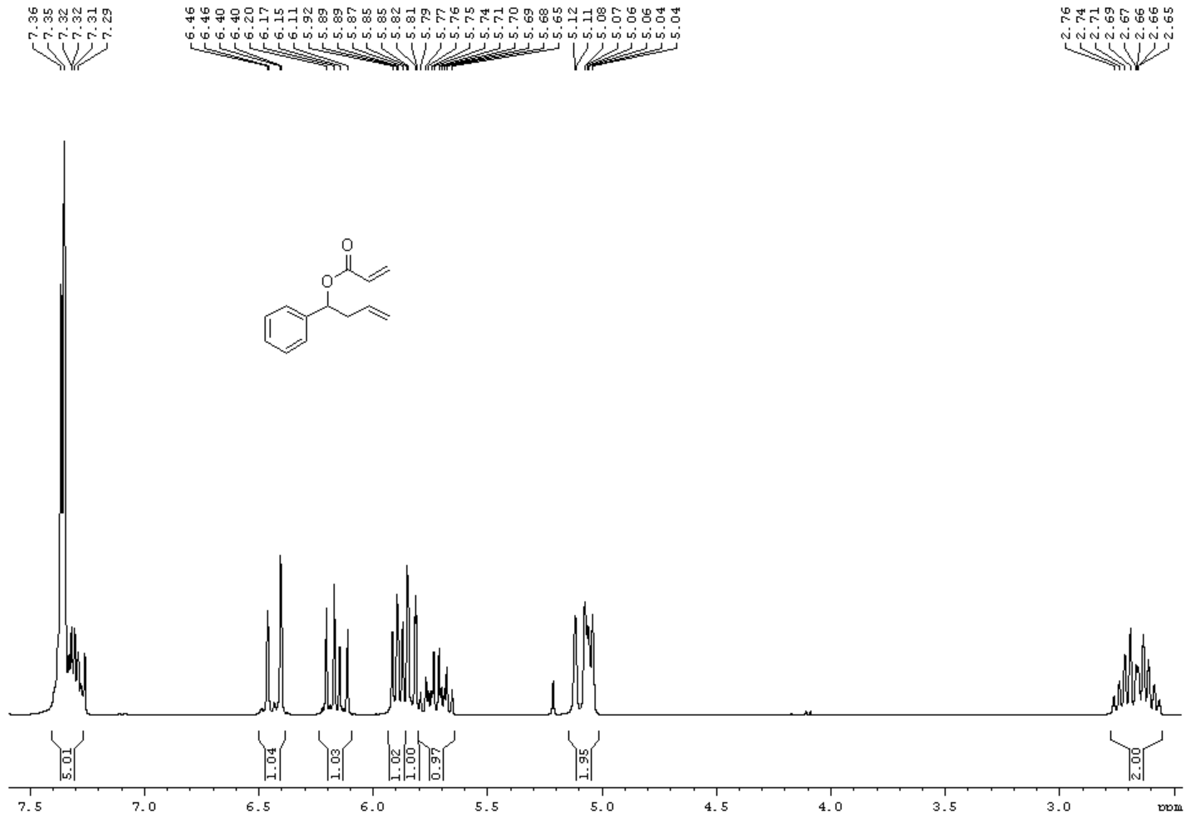

Figure S4: ^{13}C NMR spectrum of compound **3a** ($CDCl_3$, 75 MHz).

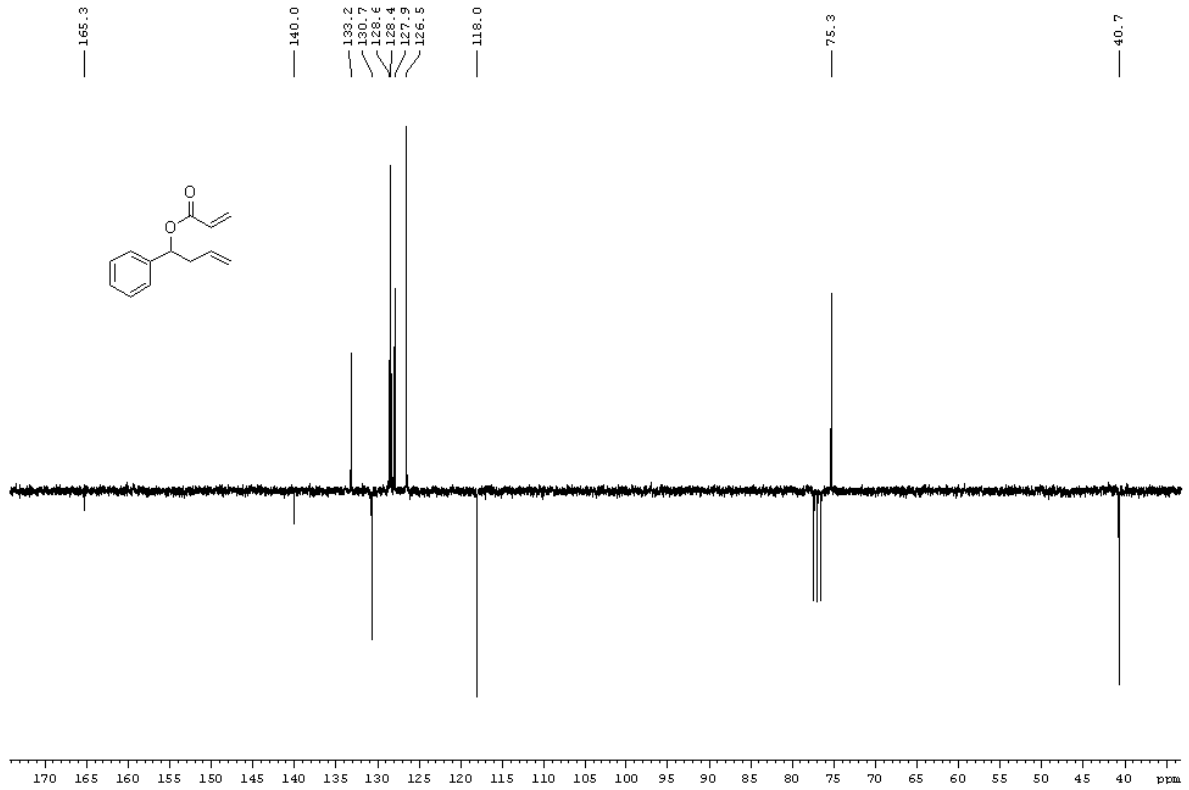

Figure S5: ^{1}H NMR spectrum of compound **3f** ($CDCl_3$, 300 MHz).

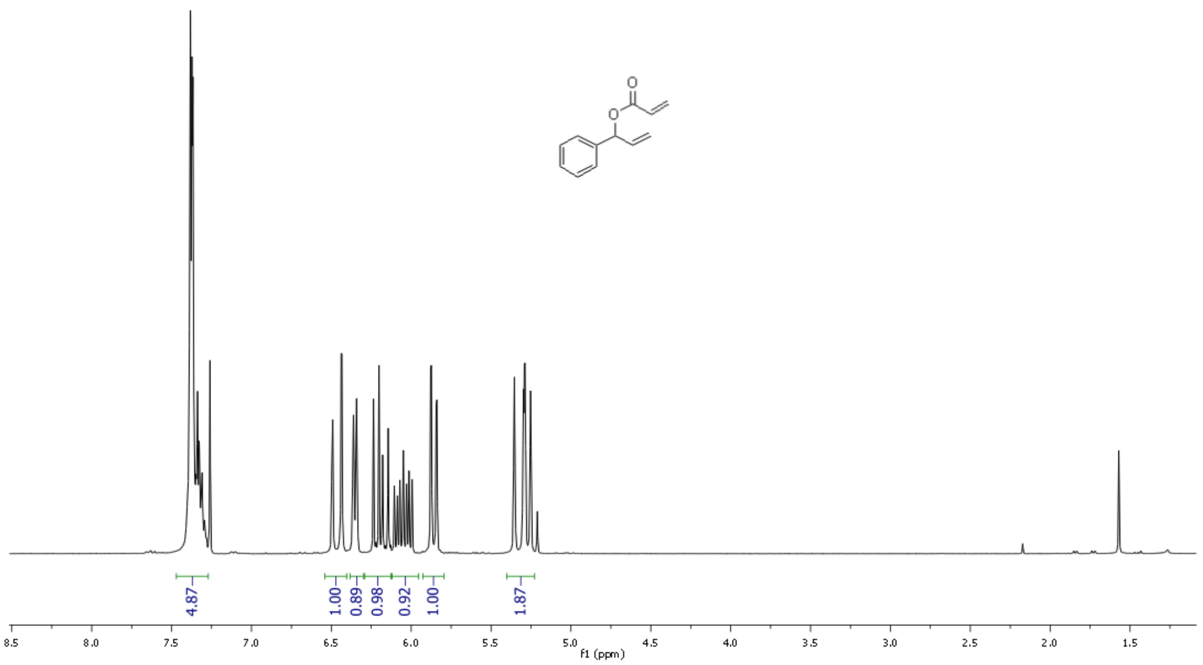

Figure S6: ^{13}C NMR spectrum of compound **3f** ($CDCl_3$, 75 MHz).

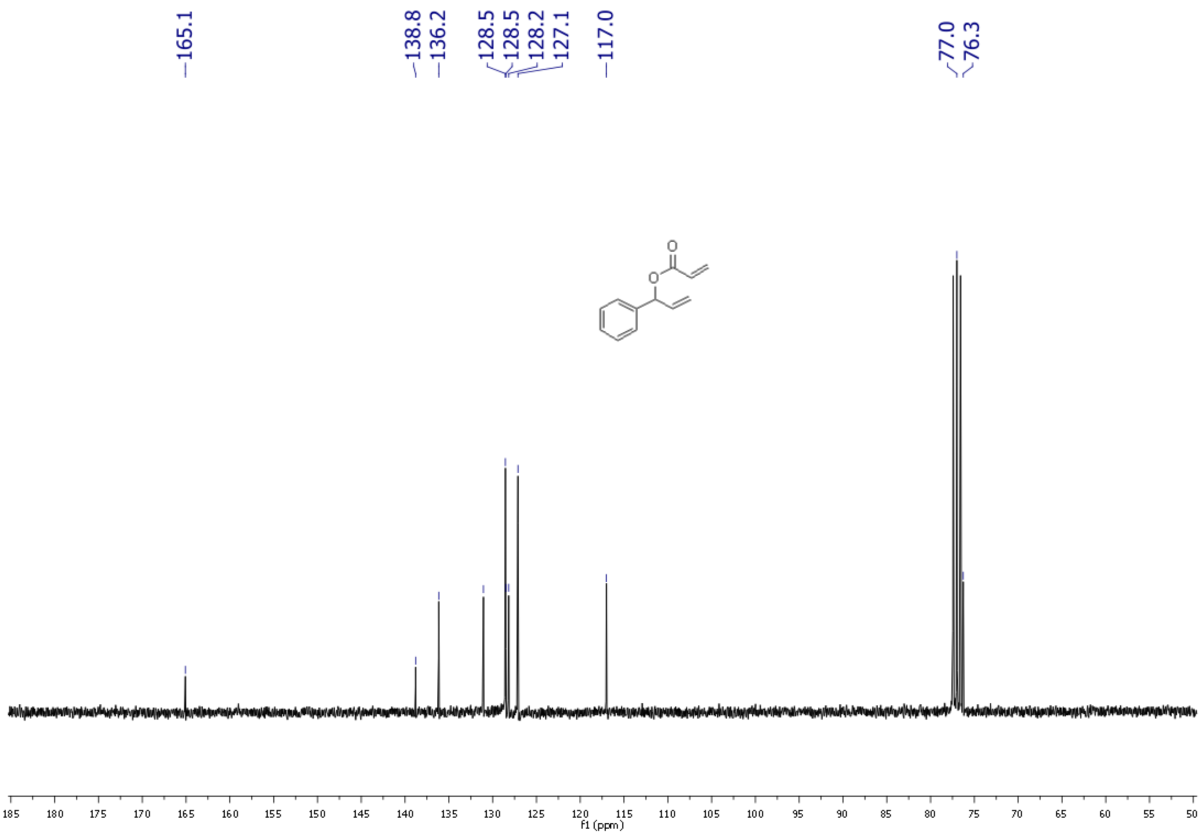

Figure S7: ^{1}H NMR spectrum of compound **4a** ($CDCl_3$, 300 MHz).

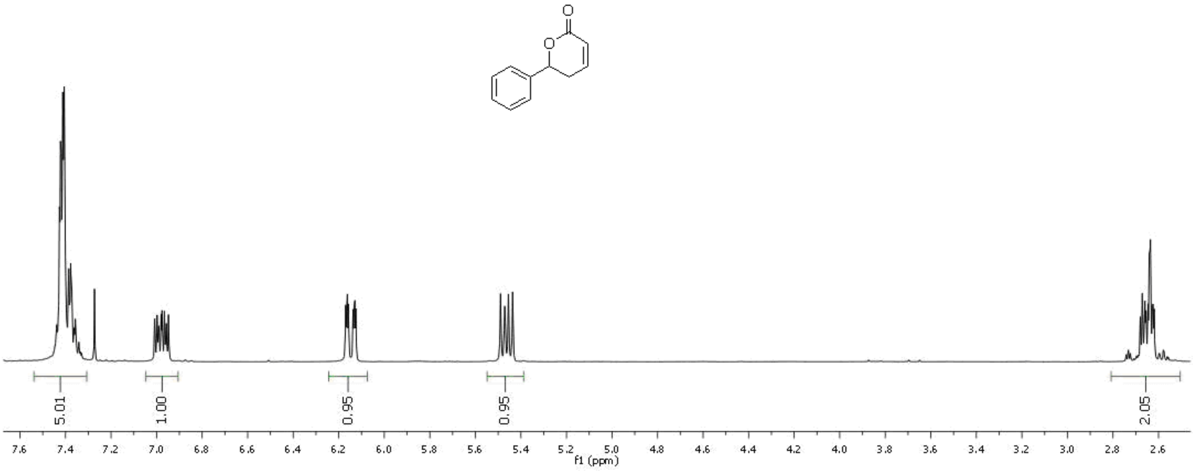

Figure S8: ^{13}C NMR spectrum of compound **4a** ($CDCl_3$, 75 MHz).

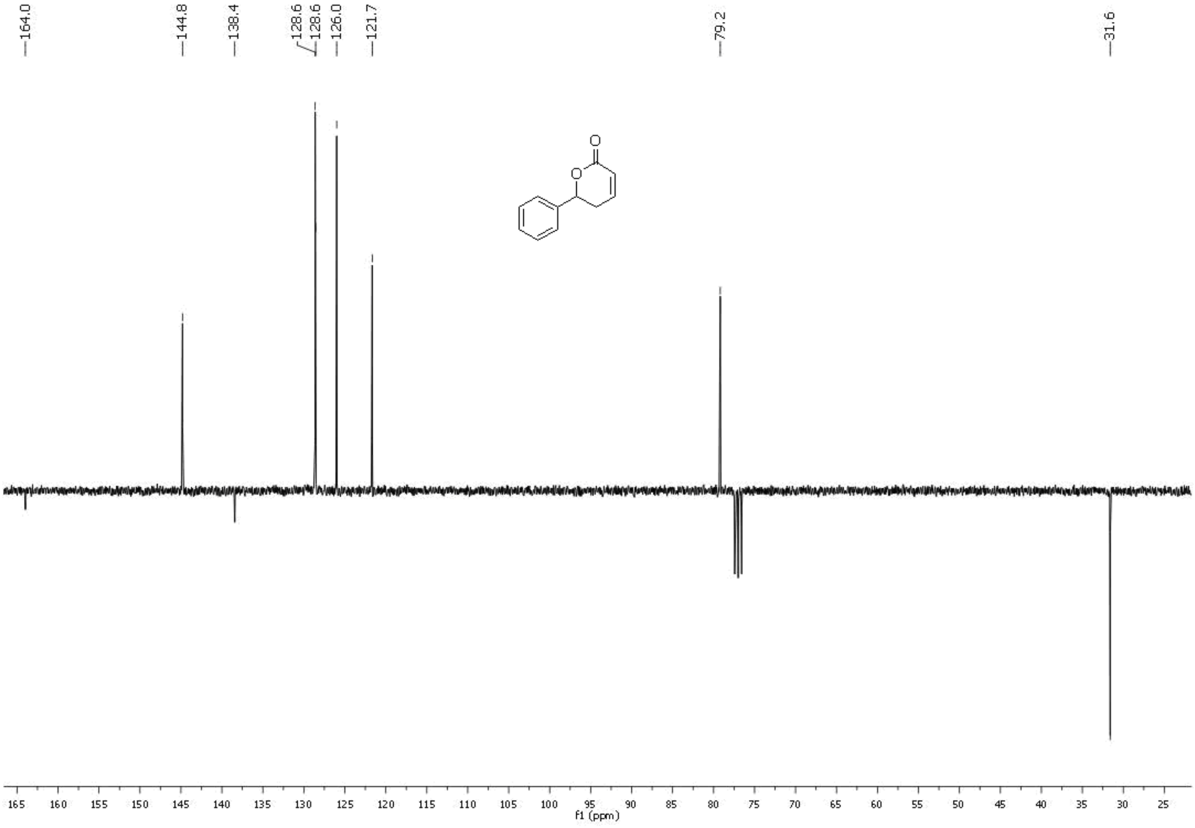

Figure S9: ^{1}H NMR spectrum of compound **4f** ($CDCl_3$, 300 MHz).

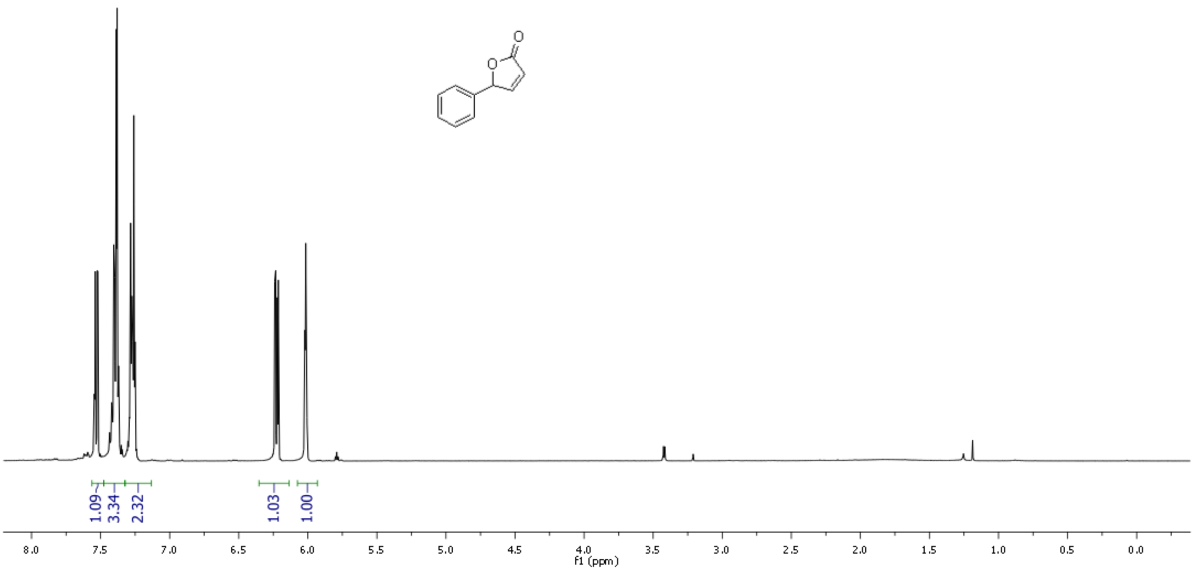

Figure S10: ^{13}C NMR spectrum of compound **4f** ($CDCl_3$, 75 MHz).

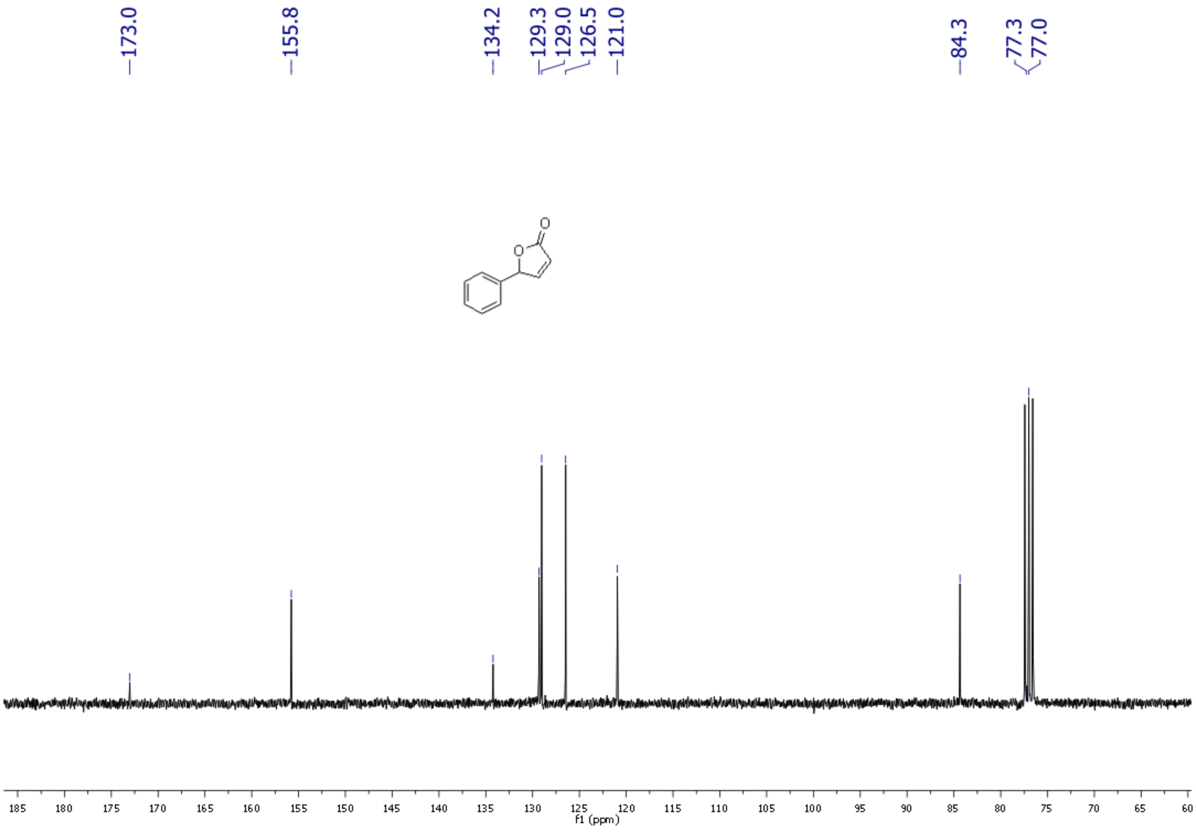

Figure S11: ^{1}H NMR spectrum of compound **7b** ($CDCl_3$, 300 MHz).

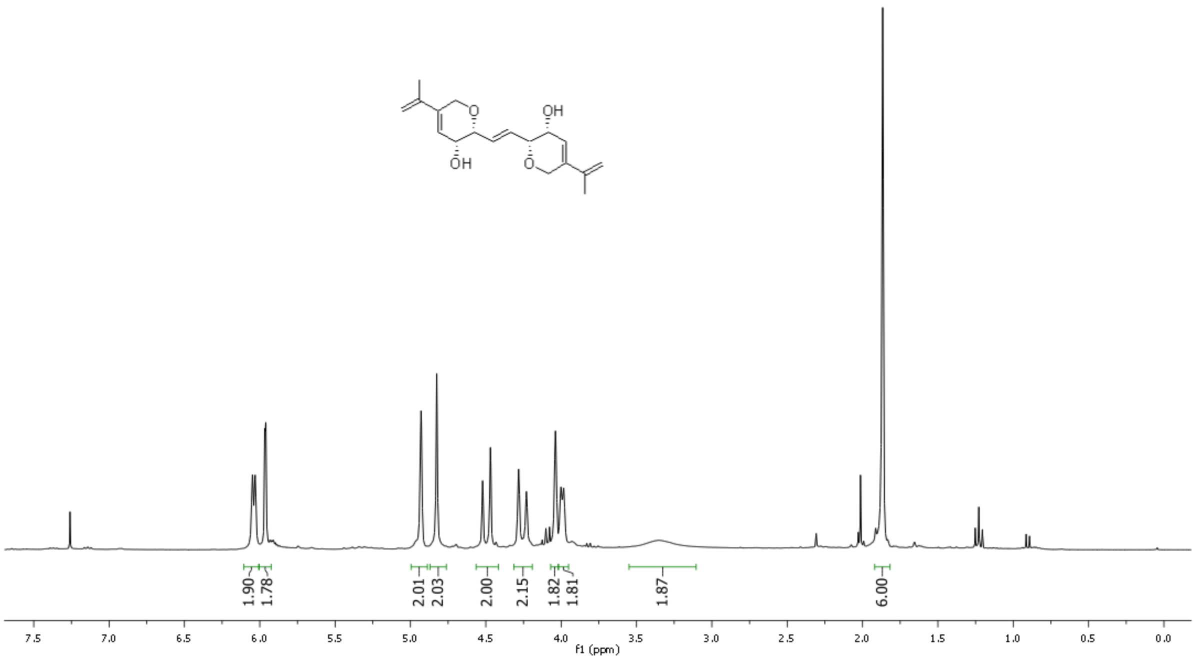

Figure S12: ^{13}C NMR spectrum of compound **7b** ($CDCl_3$, 75 MHz).

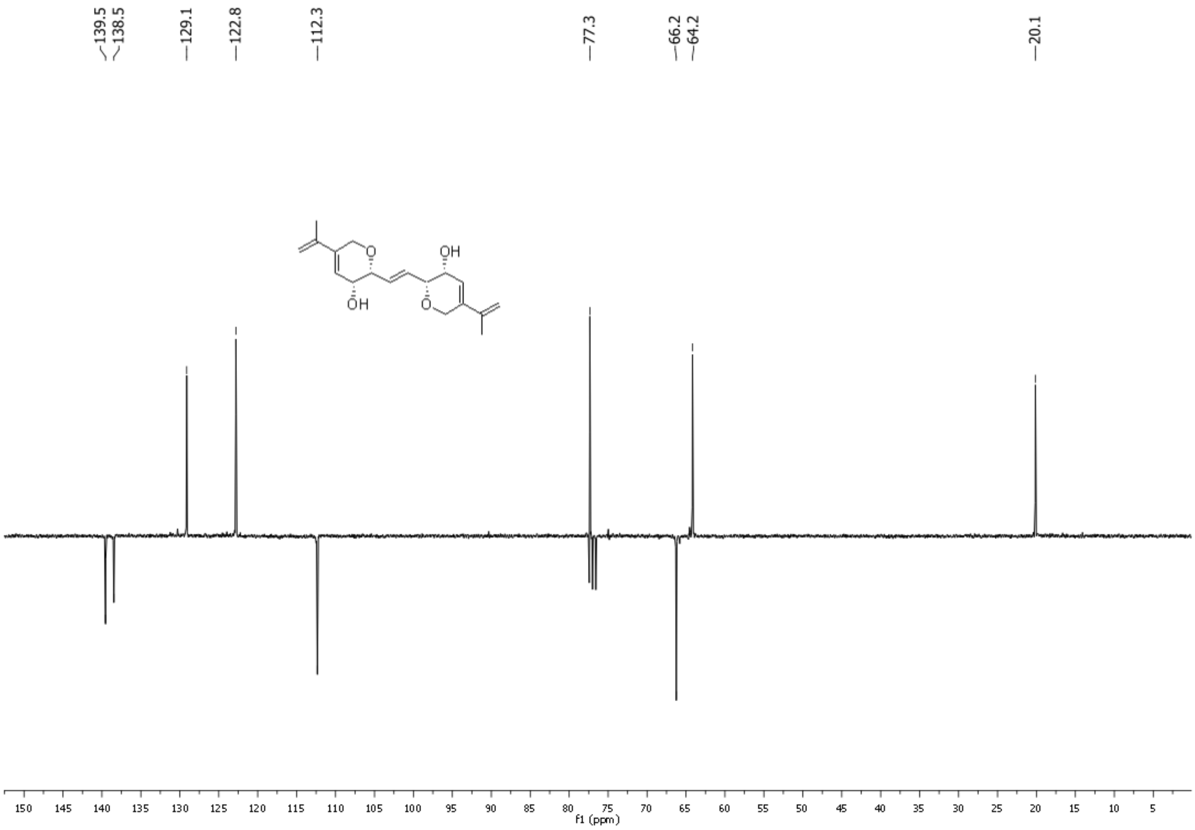

Figure S13: ^{1}H NMR spectrum of compound *rac*-**8** ($CDCl_3$, 300 MHz).

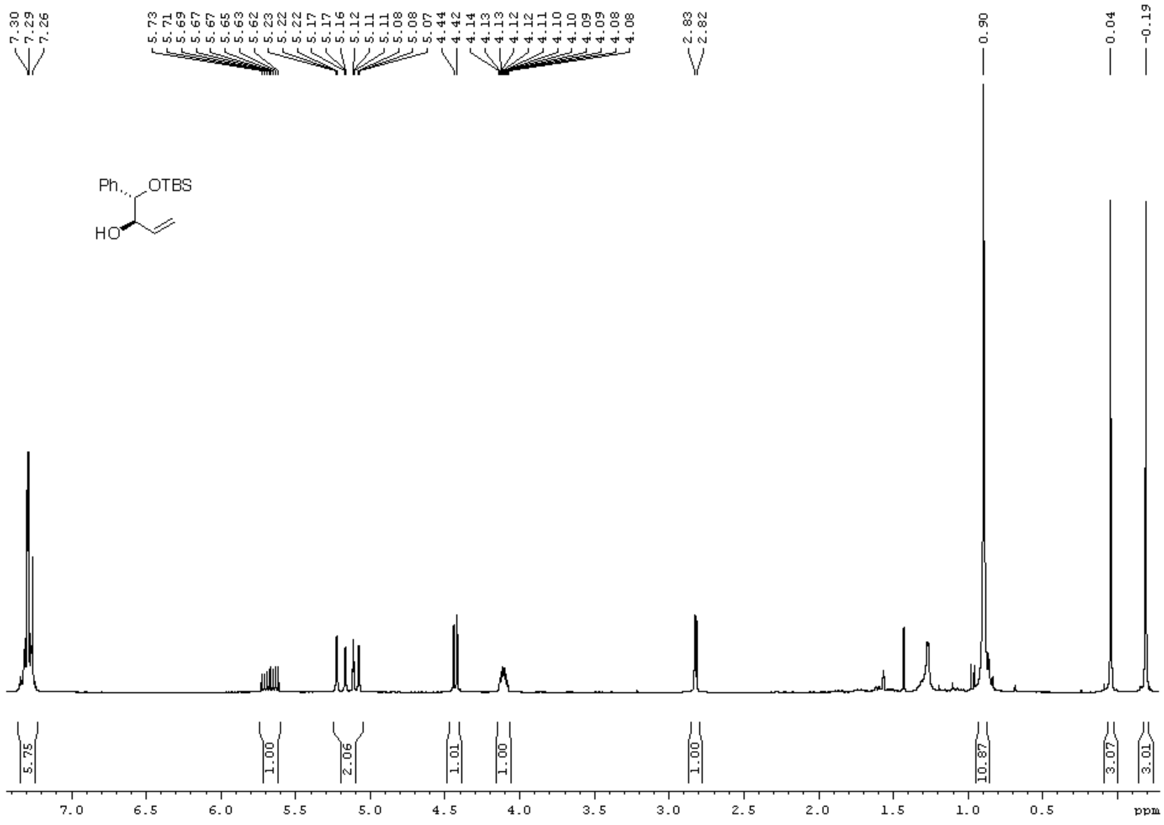

Figure S14: ^{13}C NMR spectrum of compound *rac*-**8** ($CDCl_3$, 75 MHz).

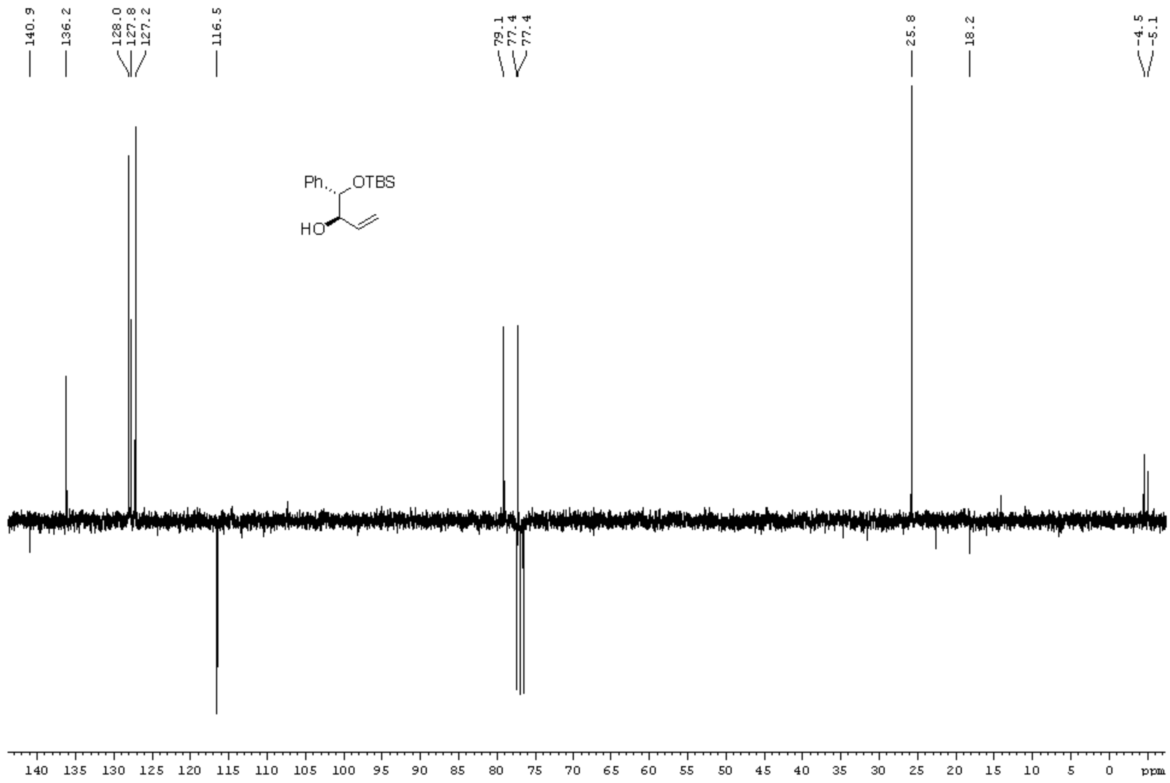

Figure S15: ^{1}H NMR spectrum of compound *rac*-**8’** ($CDCl_3$, 300 MHz).

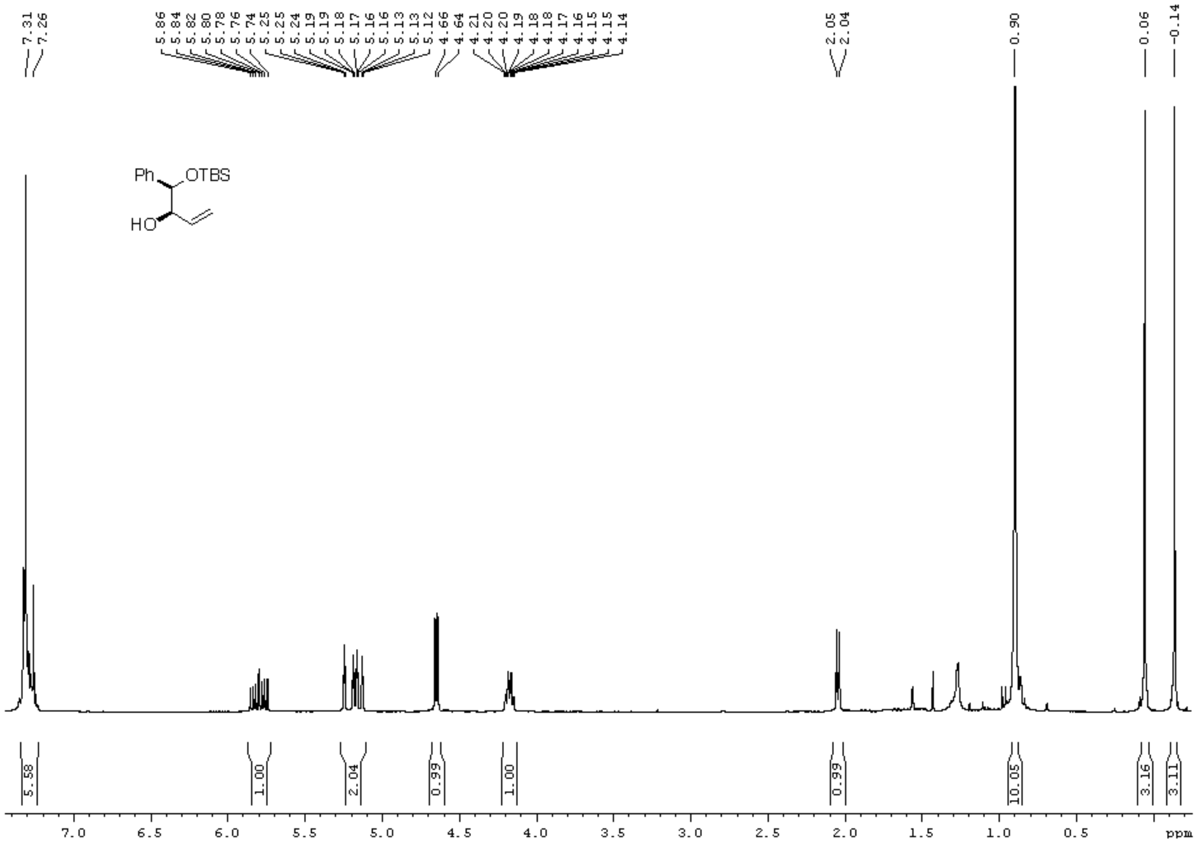

Figure S16: ^{13}C NMR spectrum of compound *rac*-**8’** ($CDCl_3$, 75 MHz).

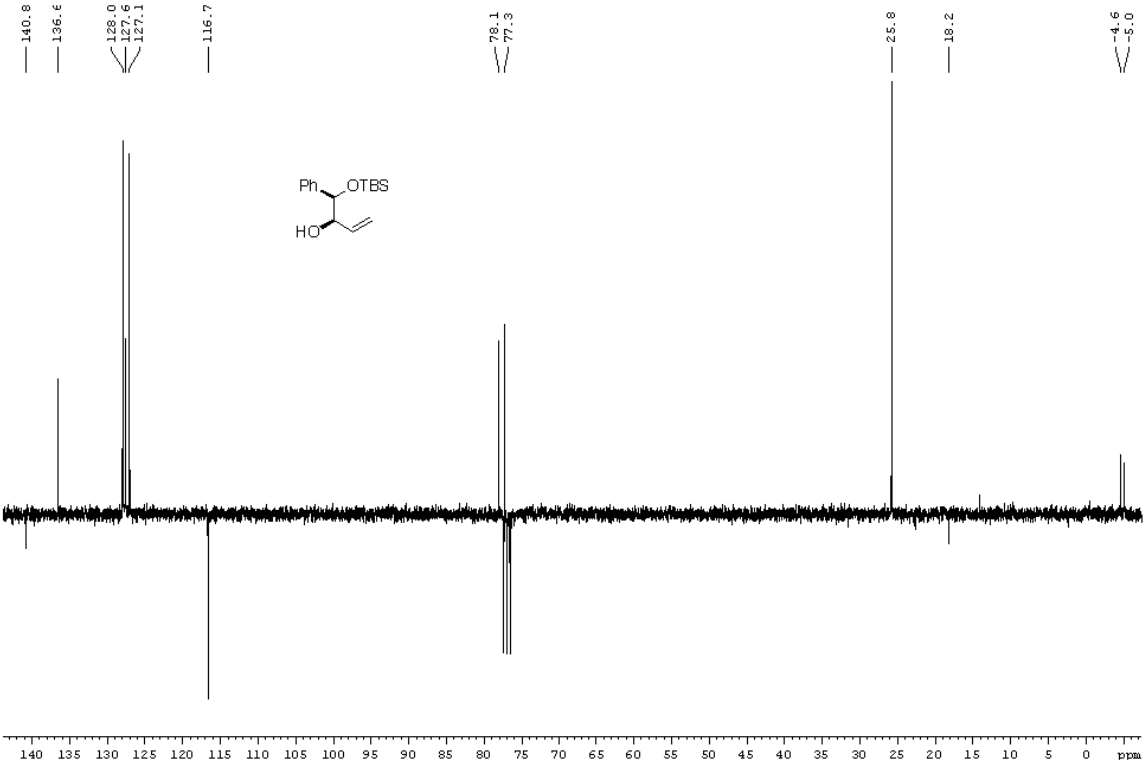

Figure S17: ^{1}H NMR spectrum of compound *rac*-**8’’** ($CDCl_3$, 300 MHz).

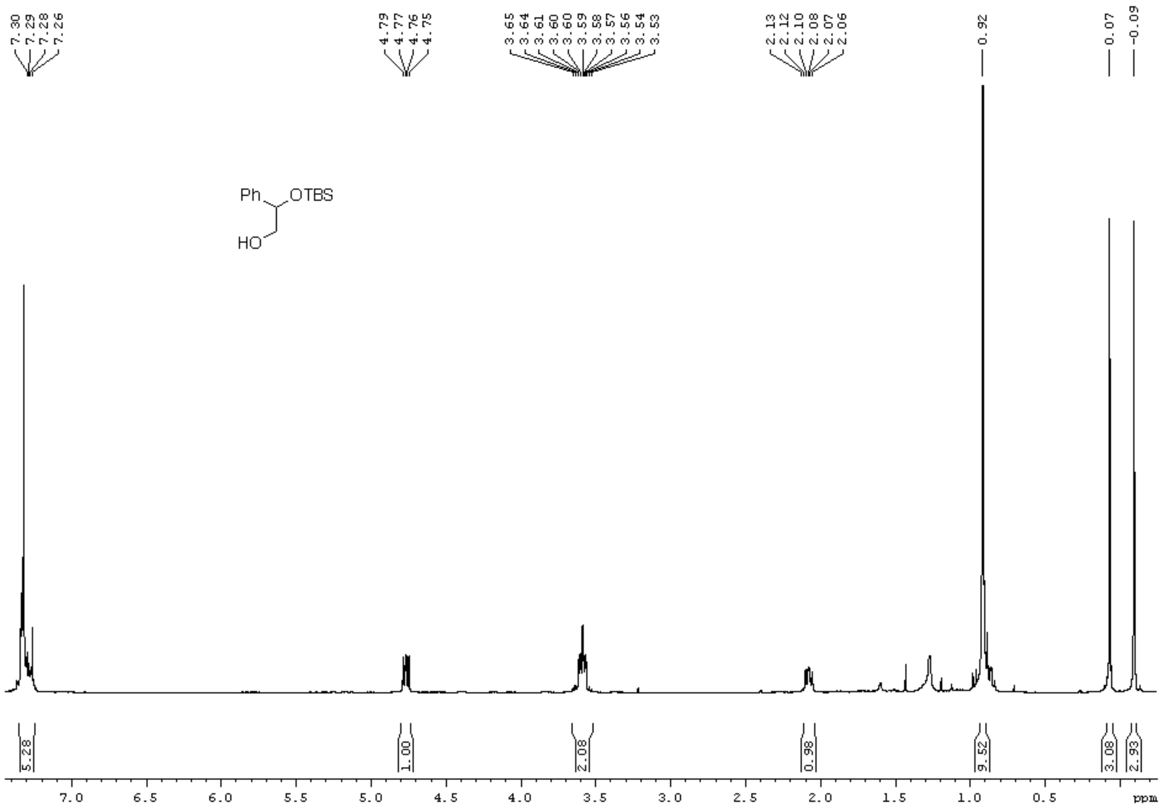

Figure S18: ^{13}C NMR spectrum of compound *rac*-**8’’** ($CDCl_3$, 75 MHz).

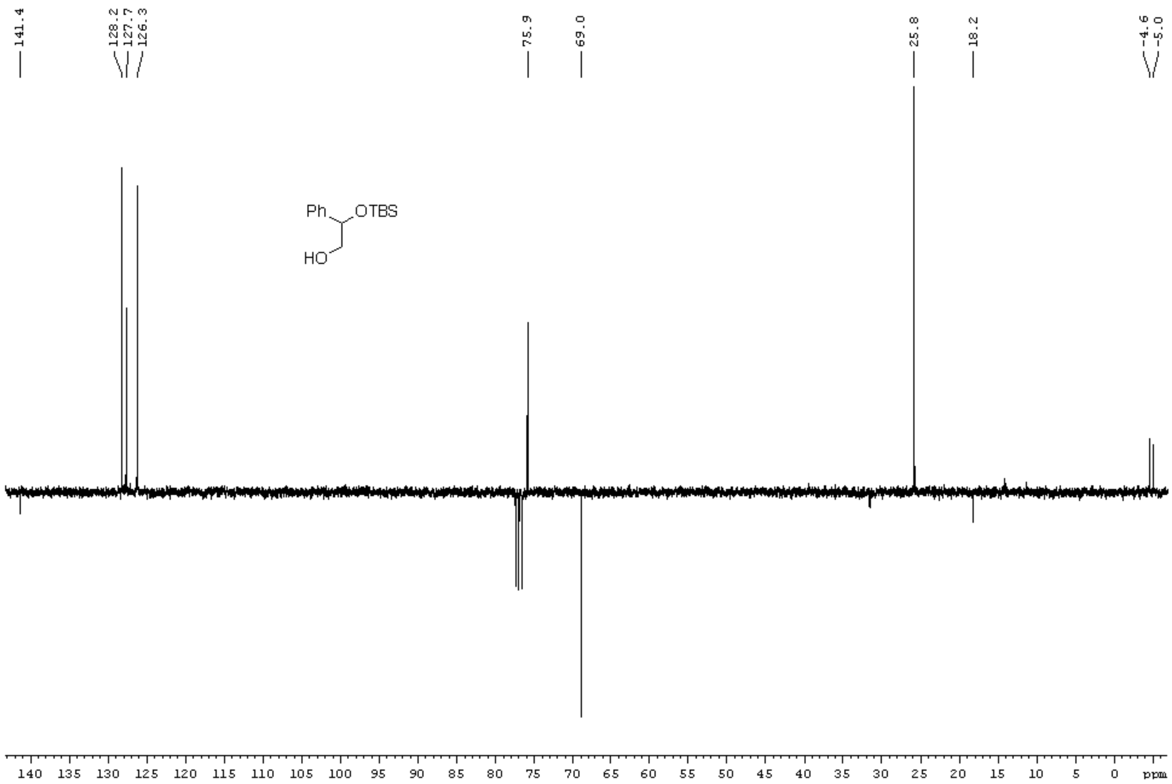

Figure S19: ^{1}H NMR spectrum of compound *rac*-**10a** ($CDCl_3$, 300 MHz).

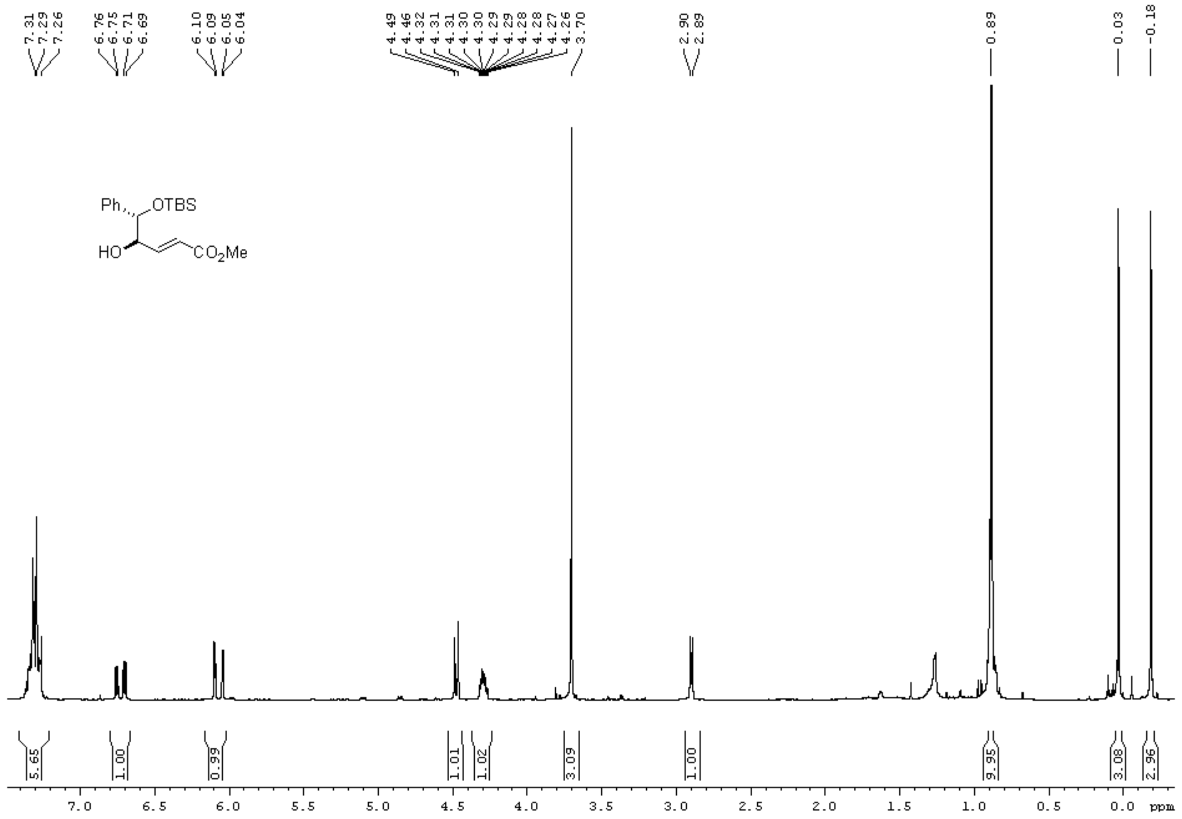

Figure S20: ^{13}C NMR spectrum of compound *rac*-**10a** ($CDCl_3$, 75 MHz).

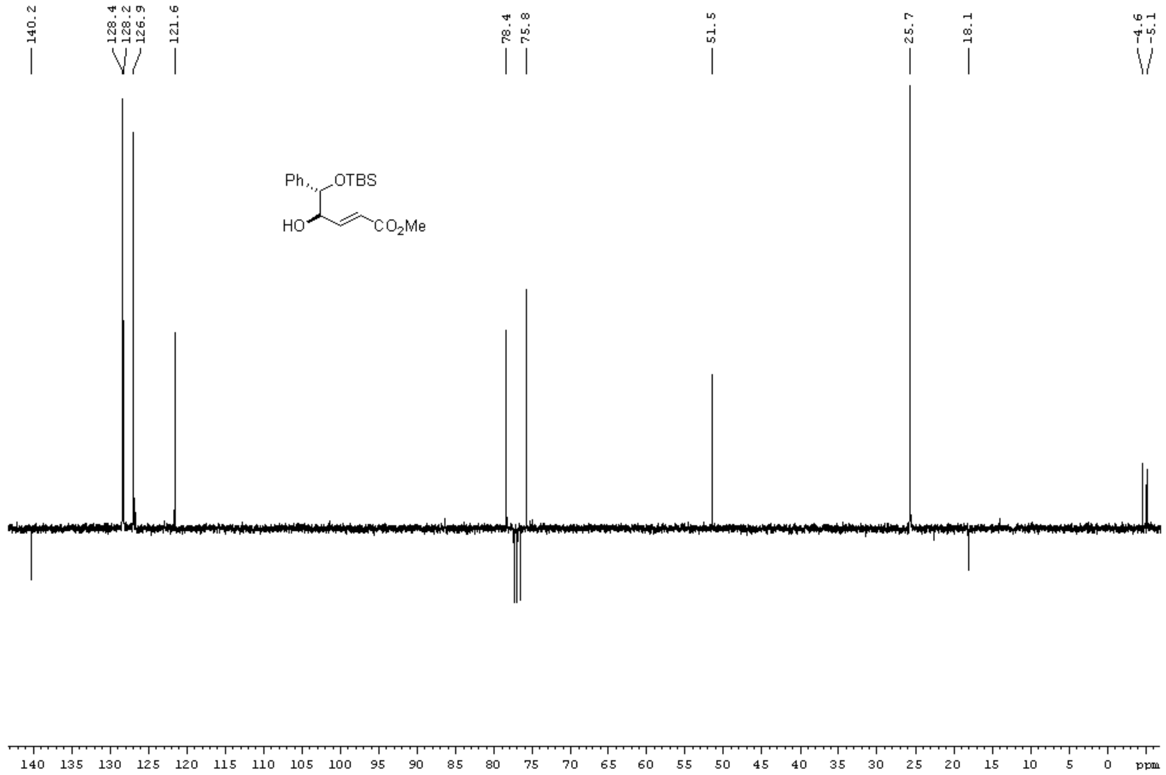

Figure S21: ^{1}H NMR spectrum of compound *rac*-**10b** ($CDCl_3$, 300 MHz).

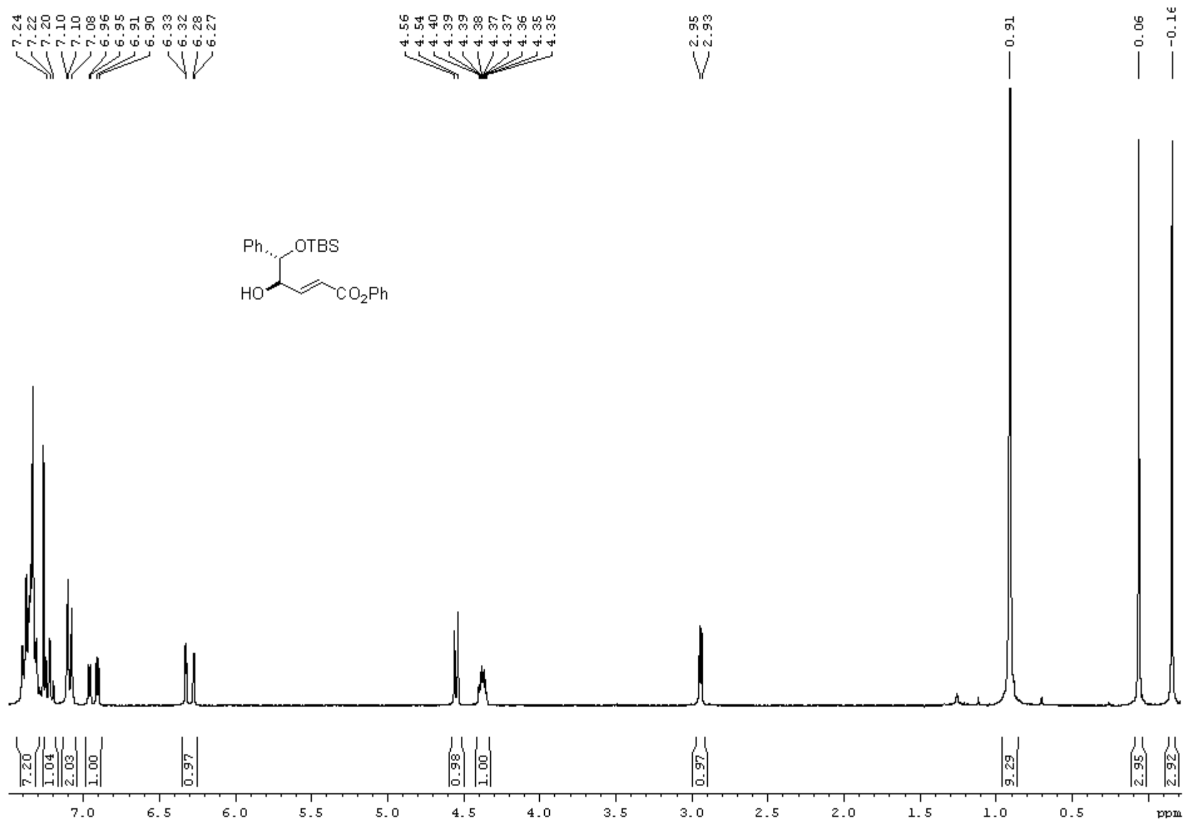

Figure S22: ^{13}C NMR spectrum of compound *rac*-**10b** ($CDCl_3$, 75 MHz).

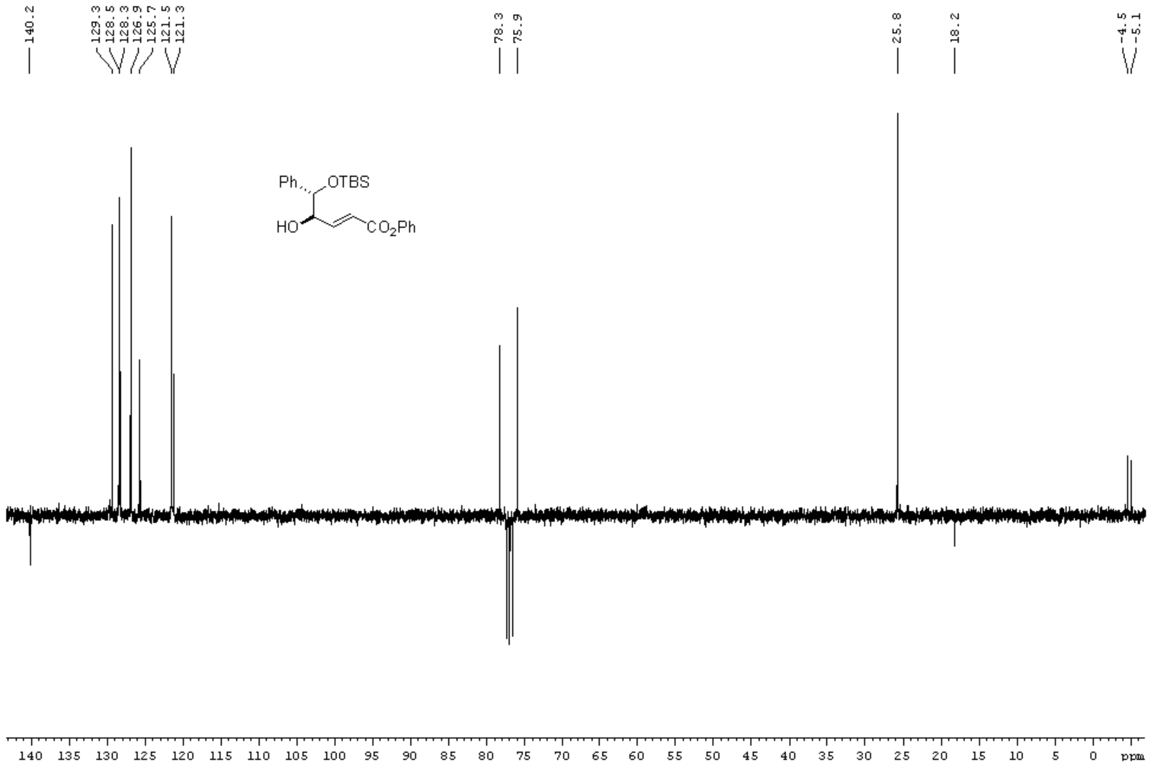

Figure S23: ^{1}H NMR spectrum of compound **11a** ($CDCl_3$, 300 MHz).

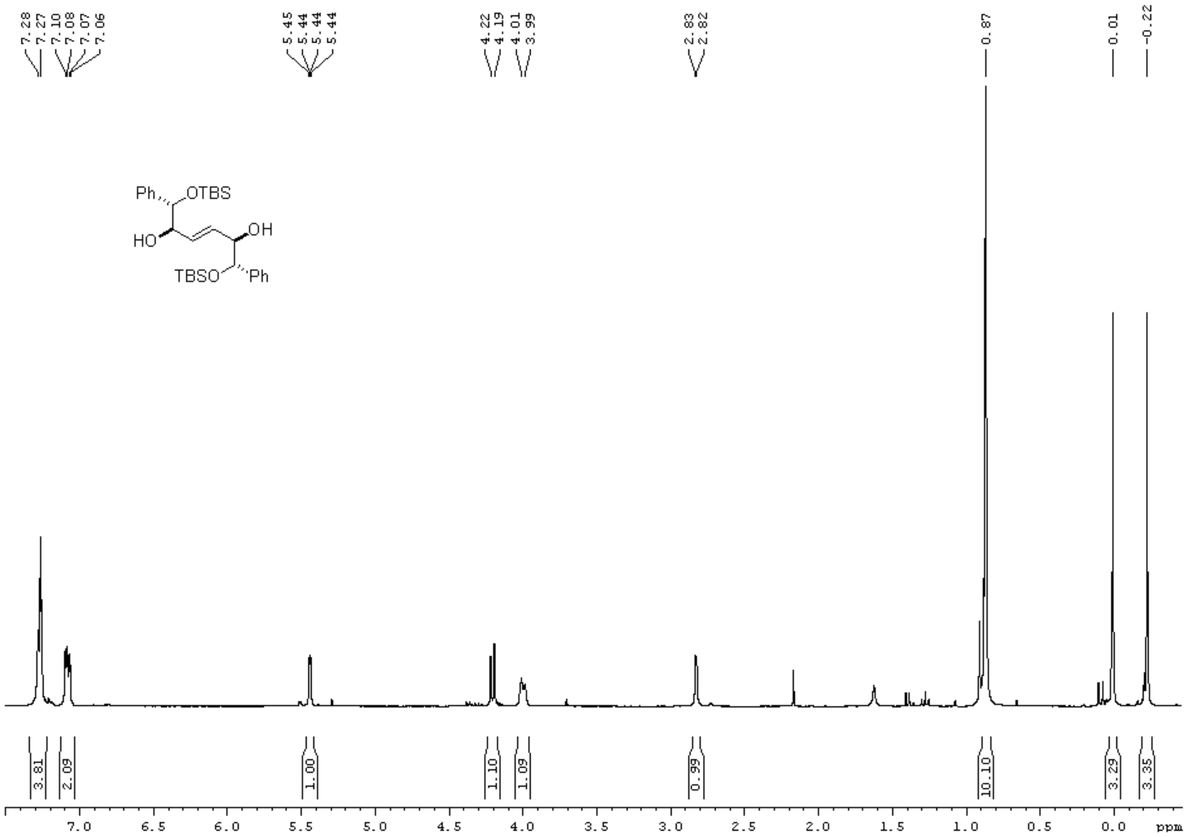

Figure S24: ^{13}C NMR spectrum of compound **11a** ($CDCl_3$, 75 MHz).

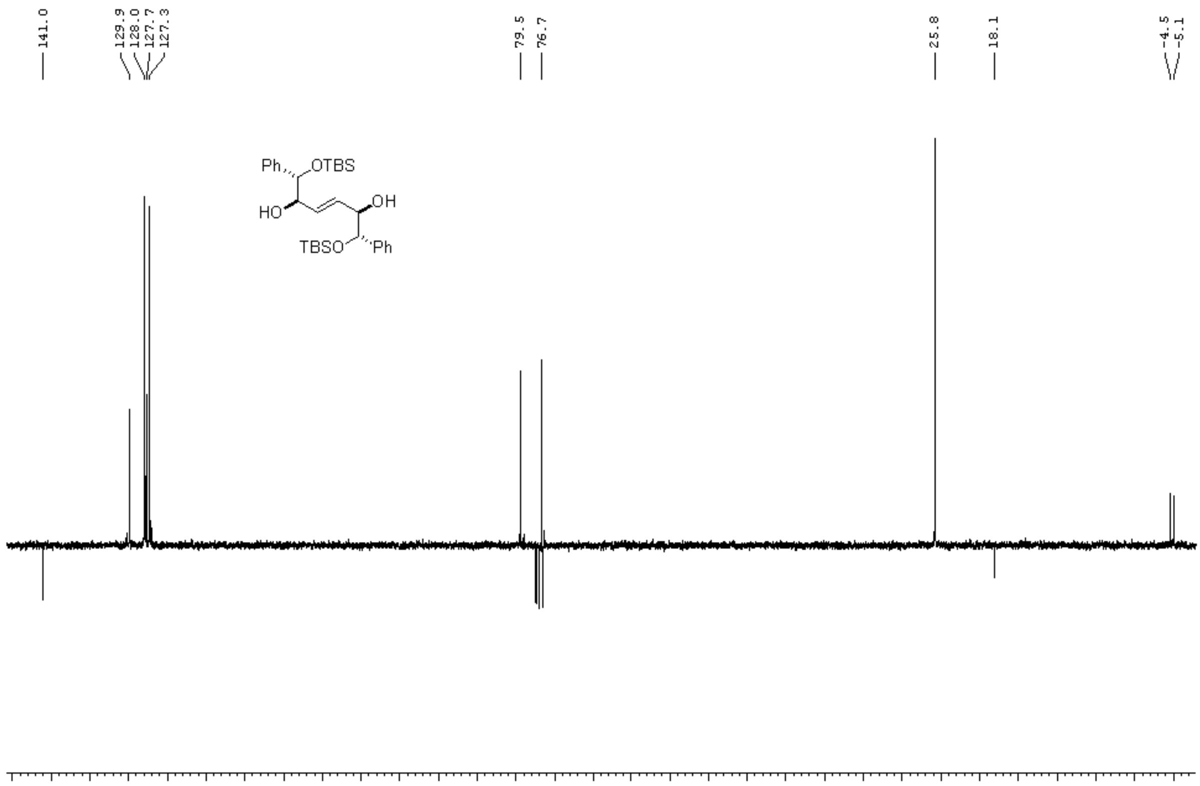

Figure S25: ^{1}H NMR spectrum of compound **11b** ($CDCl_3$, 300 MHz).

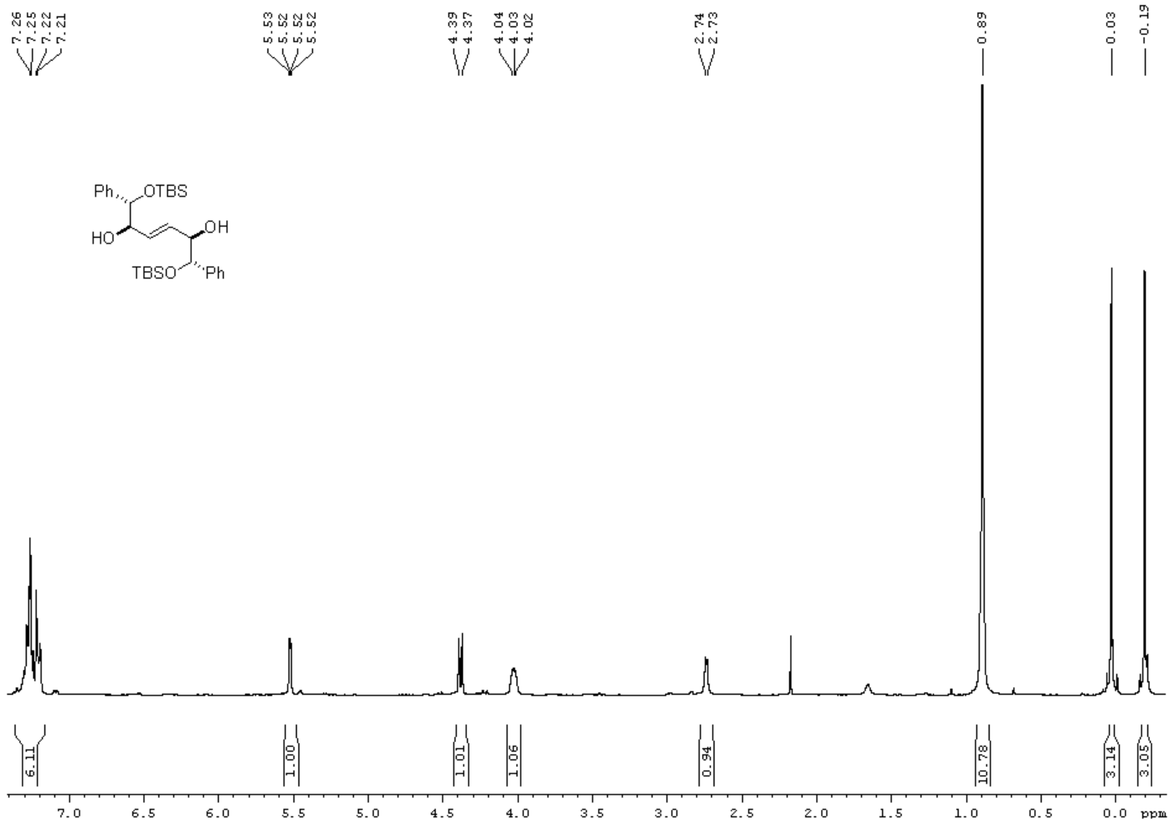

Figure S26: ^{13}C NMR spectrum of compound **11b** ($CDCl_3$, 75 MHz).

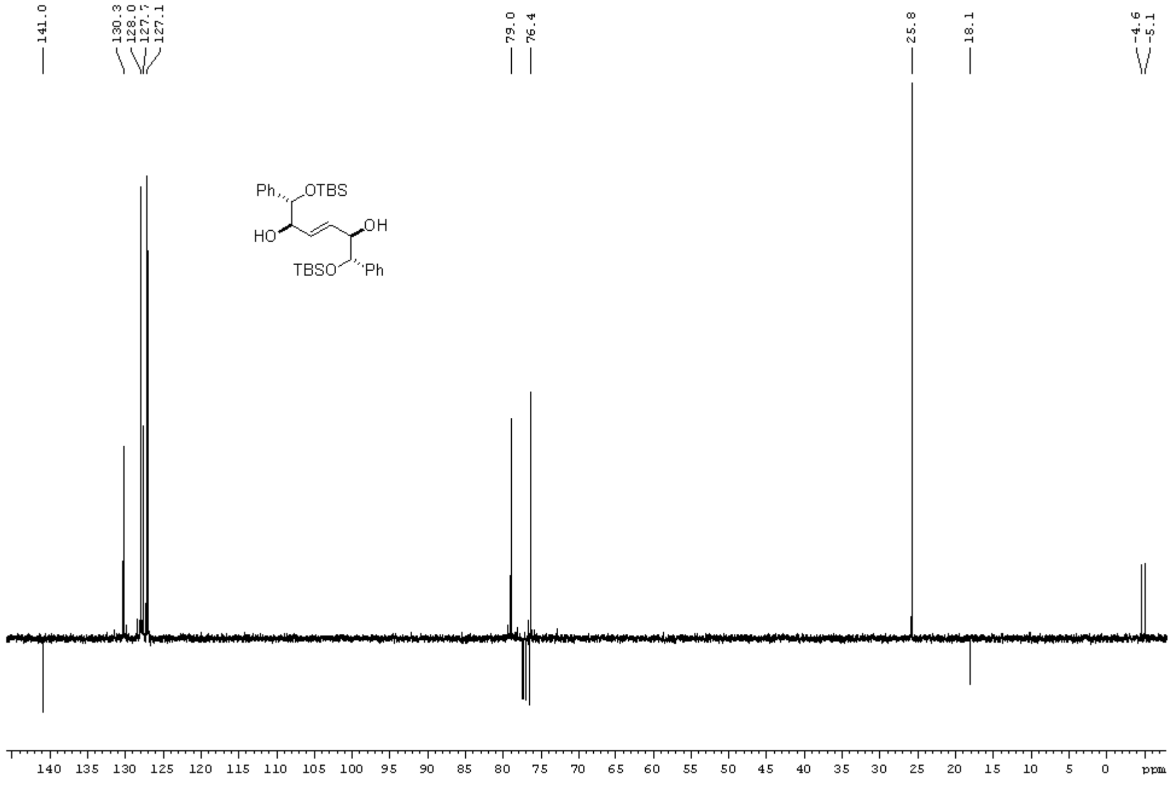

References

1. Lombardo, M.; Licciulli, S.; Trombini, C. *Tetrahedron: Asymmetry* **2004,** *15*, 289–292. doi:10.1016/j.tetasy.2003.10.026
2. de Vries, E. F. J.; Brussee, J.; van der Gen, A. *J. Org. Chem.* **1994,** *59*, 7133–7137. doi:10.1021/jo00102a047
3. Yan, F.; Moon, S.-J.; Liu, P.; Zhao, Z.; Lipscomb, J. D.; Liu, A.; Liu, H.-w. *Biochemistry* **2007,** *46*, 12628–12638. doi:10.1021/bi701370e
